# TLR4 is one of the receptors for Chikungunya virus envelope protein E2 and regulates virus induced pro-inflammatory responses in host macrophages

**DOI:** 10.3389/fimmu.2023.1139808

**Published:** 2023-04-20

**Authors:** Chandan Mahish, Saikat De, Sanchari Chatterjee, Soumyajit Ghosh, Supriya Suman Keshry, Tathagata Mukherjee, Somlata Khamaru, Kshyama Subhadarsini Tung, Bharat Bhusan Subudhi, Soma Chattopadhyay, Subhasis Chattopadhyay

**Affiliations:** ^1^ School of Biological Sciences, National Institute of Science Education and Research Bhubaneswar, Jatni, Odisha, India; ^2^ Homi Bhabha National Institute, Training School Complex, Anushaktinagar, Mumbai, Maharashtra, India; ^3^ Institute of Life Sciences, Bhubaneswar, India; ^4^ Regional Centre for Biotechnology, Faridabad, India; ^5^ School of Biotechnology, Kalinga Institute of Industrial Technology (KIIT) University, Bhubaneswar, India; ^6^ School of Pharmaceutical Sciences, Siksha O Anusandhan Deemed to be University, Bhubaneswar, Odisha, India

**Keywords:** Chikungunya virus (CHIKV), toll-like receptor 4 (TLR4), CHIKV-E2, pro-inflammatory cytokines, inflammation

## Abstract

Toll like receptor 4 (TLR4), a pathogen-associated molecular pattern (PAMP) receptor, is known to exert inflammation in various cases of microbial infection, cancer and autoimmune disorders. However, any such involvement of TLR4 in Chikungunya virus (CHIKV) infection is yet to be explored. Accordingly, the role of TLR4 was investigated towards CHIKV infection and modulation of host immune responses in the current study using mice macrophage cell line RAW264.7, primary macrophage cells of different origins and *in vivo* mice model. The findings suggest that TLR4 inhibition using TAK-242 (a specific pharmacological inhibitor) reduces viral copy number as well as reduces the CHIKV-E2 protein level significantly using p38 and JNK-MAPK pathways. Moreover, this led to reduced expression of macrophage activation markers like CD14, CD86, MHC-II and pro-inflammatory cytokines (TNF, IL-6, MCP-1) significantly in both the mouse primary macrophages and RAW264.7 cell line, *in vitro*. Additionally, TAK-242-directed TLR4 inhibition demonstrated a significant reduction of percent E2-positive cells, viral titre and TNF expression in hPBMC-derived macrophages, *in vitro*. These observations were further validated in TLR4-knockout (KO) RAW cells. Furthermore, the interaction between CHIKV-E2 and TLR4 was demonstrated by immuno-precipitation studies, *in vitro* and supported by molecular docking analysis, *in silico.* TLR4-dependent viral entry was further validated by an anti-TLR4 antibody-mediated blocking experiment. It was noticed that TLR4 is necessary for the early events of viral infection, especially during the attachment and entry stages. Interestingly, it was also observed that TLR4 is not involved in the post-entry stages of CHIKV infection in host macrophages. The administration of TAK-242 decreased CHIKV infection significantly by reducing disease manifestations, improving survivability (around 75%) and reducing inflammation in mice model. Collectively, for the first time, this study reports TLR4 as one of the novel receptors to facilitate the attachment and entry of CHIKV in host macrophages, the TLR4-CHIKV-E2 interactions are essential for efficient viral entry and modulation of infection-induced pro-inflammatory responses in host macrophages, which might have translational implication for designing future therapeutics to regulate the CHIKV infection.

## 1 Introduction

Since the first report in 1952, the Chikungunya virus (CHIKV) (Family: Togaviridae; Genus: Alphavirus) has been considered a global public threat over the years. Two massive outbreaks in the last two decades (2004 and 2013) across different regions of the globe emphasize the severity and re-emerging nature of Chikungunya. One of the major governing factors for these repeated outbreaks are mainly unhygienic densely populated habitat with ineffective mosquito control capacity as Chikungunya in mosquito-borne (*Aedes* sp.) disease. Other associated factors are favorable climate for mosquito breeding, lack of available vaccines and proper medications (1, 2).

The pathophysiological manifestations of Chikungunya can be classified into three stages, namely, acute, sub-acute and chronic. The major symptoms of the acute stage are mainly high fever, polyarthralgia, headache, loss of appetite and rashes. The symptoms may last up to 3 months for the sub-acute stage. Although the acute stage has less severity, it may bring severe complications in neonates, pregnant women, patients suffering from comorbidities and aged people (over 65 years). The reported complications are failure of either neuronal, cardiovascular, renal, or respiratory systems. The chronic stage of infection may affect around 40% of the patients and the major symptoms are chronic arthralgia, myalgia, long term fatigue which might lead to permanent physical disability (1, 3, 4).

The mechanistic view on CHIKV entry in the host is not well understood till date. However, several entry pathways, for example, the clathrin-mediated pathway, epidermal growth factor receptor substrate 15 (Eps15)-dependent pathway and macropinocytosis have been experimentally demonstrated to be associated with CHIKV attachment and entry in the host (5–7). For CHIKV attachment, cell surface glycosaminoglycans (GAG), glycoprotein T-cell immunoglobulin and mucin 1 (TIM-1), TIM-4, Axl, C-type calcium-dependent lectin DC-SIGN (DC-specific intercellular adhesion molecule-3-grabbing non-integrin) and prohibitin (PHB) 1 and 2 were found as interaction and attachment factors in the host (8–17). Recently, a cell adhesion molecule, Mxra8 has been found to block CHIKV infection in presence of an anti-Mxra8 monoclonal antibody, although the absence of functional Mxra8 could not completely block CHIKV infection *in vitro* and *in vivo* (18). Therefore, Mxra8 acts as one of the enhancers for CHIKV attachment and internalization process into the host cell.

Several clinical and experimental studies have revealed that the Chikungunya virus (CHIKV) infection leads to the profound production of pro-inflammatory cytokines and chemokines such as tumor necrosis factor (TNF), interleukin (IL)-6, 4, 1β and 12 in human as well as in mouse macrophages *via* p38 and Jun N-terminal protein kinase (JNK)-mitogen-activated protein kinase (MAPK) mediated pathway, which may aggravate host immune system towards CHIKV infection mediated fever (CHIKF) and polyarthralgia (19–22). However, the initial pathways behind CHIKV-driven pro-inflammatory responses are still unexplored. Interestingly, the role of toll like receptor 4 (TLR4) has been critically investigated for mediating inflammatory responses in various cases of microbial infections, immune regulation in cancer and autoimmunity (23–25). TLR4 has also been well reported to induce massive pro-inflammatory responses upon binding of lipid A region of lipopolysaccharide (LPS), a cell wall component of Gram-negative bacteria (26). Moreover, the functional association of TLR4 is well established for other pro-inflammatory clinical abnormalities such as inflammatory bowel disease (IBD) and necrotizing enterocolitis (NEC) (27, 28). To establish the role of TLR4 in various *in vivo* inflammatory conditions such as mice sepsis model or LPS-induced lung injury model, a cyclohexene derivative molecule, TAK-242, has been used as a specific blocker of TLR4-dependent inflammation (24, 29). Furthermore, TLR4-dependent viral entry and infection progression of respiratory syncytial virus (RSV) has been described in mice model (30). Recently, several viral structural proteins are proposed to act as potential ligands for TLR4 activation (31, 32).

CHIKV-induced host cell activation and a rise in associated pro-inflammatory responses are already reported by us and others (19, 22, 33). Earlier studies have revealed that the pro-inflammatory cytokines along with MAPKs are induced during CHIKV infection in the host macrophages (19, 22). Since TLR4 activation could be connected with TNF response and MAPK activation (34, 35), the possible interaction of TLR4 with CHIKV infection along with subsequent regulation of host immune responses, if any, needs to be explored. Hence, it has been hypothesized that TLR4 might be pivotal to regulate CHIKV infection and associated host immune responses. Accordingly, in the current study, the probable role of TLR4 has been investigated in CHIKV infection, inflammation and modulation of host immune responses using different *in vitro* models, *in silico* studies and *in vivo* mice model.

## 2 Materials and methods

### 2.1 Cells, virus and reagents

The RAW264.7 (ATCC^®^ TIB-71™), BALB/c and C57BL/6 mice-derived peritoneal monocyte-macrophage cells were maintained in complete RPMI media consisting RPMI-1640 (Gibco, USA), supplemented with antibiotic-antimycotic solution, L-glutamine (HiMedia Laboratories Pvt. Ltd, MH, India) and 10% heat-inactivated Fetal bovine serum (Gibco, USA) at 37°C in a humidified incubator with 5% CO_2_. The TLR4KO RAW (RAW-Dual KO™-TLR4; catalog number: rawd-kotlr4, Invivogen, USA) (36) and the Vero Cells were maintained in DMEM (catalog number: 11965-092; Gibco, USA) supplemented with 10% FBS, L-glutamine and antibiotic-antimycotic solution. The CHIKV-Indian Strain (IS) (accession no- EF210157.2), anti-CHIKV-E2 antibody and Vero cells were kind gifts from Dr. M.M. Parida, DRDE, Gwalior, India. The anti-CHIKV-E1 antibody was a kind from Dr. T.K. Chowdary, NISER, Bhubaneswar, India. TAK-242 (catalog no: 614316-5MG**)**, a well-cited TLR4 inhibitor was purchased from Merck Millipore, USA (24, 34, 37). The antibodies against CD86 (Fluorochrome: APC; Catalogue number: 17-0862-82) and MHC-II (Fluorochrome: PE; Catalogue number: 12-5321-82) were purchased from eBiosciences, USA. PerCP-Cy5.5 conjugated CD14 antibody (catalog number: 560638) was purchased from BD Biosciences, USA. The unconjugated antibodies against p-NF-κB p65 (Catalogue number: 3031), total p38 (catalog number: 9212), phosphorylated p38 (catalog number:9211), total SAPK-JNK (catalog number:9252) and phosphorylated SAPK-JNK (catalog number: 4668) proteins were bought from Cell Signaling Technology (Denver, USA). Alexa fluor (AF)-647 conjugated TLR4-MD2 monoclonal antibody (clone Number: MTS510, catalog number: NBP2-24865AF647), used in flow cytometry, was purchased from Novus Biologicals (Littleton, Colorado, USA). The TLR4 polyclonal antibody (catalog number: 48-2300), used in co-immunoprecipitation and Western Blot, was purchased from Invitrogen (Carlsbad, USA). Fluorochrome (AF488/AF647) conjugated anti-mouse and rabbit secondary antibodies (used for flow cytometry) and HRP-conjugated anti-mouse and rabbit secondary antibodies (used in Western Blot and co-immunoprecipitation analysis) were purchased from Invitrogen, USA. The GAPDH (catalog number: 10-10011) and β-actin (catalog number: 11-13012) antibodies were bought from Abgenex India Pvt. Ltd, Bhubaneswar, India.

### 2.2 hPBMC isolation

Human blood was drawn from healthy donors following the guidelines of the Institutional Ethics Committee, NISER, Bhubaneswar (NISER/IEC/2022-04). The procedure for generating myeloid adherent cells from human peripheral blood mononuclear cells (hPBMC) was followed as described elsewhere with little modifications (38–41). Briefly, circulating monocytes were enriched by 2 h adherence after Hi-Sep LSM (catalog number: HiSep LSM™ 1077‐ LS001; HiMedia Laboratories Pvt Ltd, India) based density gradient-centrifugation according to the manufacturer’s instructions. The adherent cells were cultured in RPMI-1640 supplemented with 10% FBS, antibiotic-antimycotic solution and L-glutamine for 3–5 days. The adherent cells obtained after 96 h were of monocyte-macrophage lineages (more than 97%) as found enriched with CD14^+^CD11b^+^ population (42, 43). The monocyte-macrophage cells derived from hPBMC were seeded in 12 well plates (Thermo Fischer, USA) at a density of 0.8x10^6^ cells/well. After 24 h of seeding, pre-incubation was carried out for 3 h with 1 µM of TAK-242, followed by CHIKV infection with MOI 5 for 2 h (19). The infected cells were harvested at 8 hours post-infection (hpi) and downstream experiments were conducted.

### 2.3 Cell viability assay

The working concentrations of TAK-242 in different host macrophage systems were determined using either the AnnexinV-7-AAD-based method (Annexin V: PE Apoptosis detection kit I, catalog number: 559763; BD Biosciences, USA) or MTT assay-based method (EZcount™ MTT cell assay kit, catalog number: CCK-003-2500; HiMedia laboratories Pvt. Ltd, India) as per manufacturer’s protocol.

### 2.4 LPS induction in RAW264.7 cells

The RAW264.7 cells were induced with LPS as per earlier reports with required modifications (44). Around 4.5x10^6^ cells were seeded per 90 mm cell culture dishes (Genetix Biotech Asia Pvt Ltd, India) for 16-18 h. The cells were washed with 1X PBS (RT) twice and pre-incubated with either DMSO or 1μM TAK-242 for 3 h. Next, the cells were treated with 500 ng/mL of LPS (catalog number: L5293-2ML, Sigma-Aldrich, Germany) for 6 h. Finally, the cells were scraped using a sterile cell scraper (Genetix, India) with 1X PBS and processed for downstream experiments.

### 2.5 CHIKV infection

The RAW264.7 cell line, TLR4KO RAW cell line, BALB/c and C57BL/6 mice-derived peritoneal monocyte-macrophages were infected with CHIKV-IS as reported earlier with minute modifications (19, 22, 40, 41, 45, 46). Briefly, 4.5x10^6^ cells were seeded in 90 mm dishes and allowed to grow for 16-18 h. Next, the cells were washed with 1X PBS 2 times and pre-incubated with either TAK-242 or DMSO for 3 h. For TAK-242 treated conditions, the cells were incubated with 0.5 and/or 1 µM concentrations of TAK-242 for 3 h before infection, during infection and post-infection. After pre-incubation, the cells were washed followed by CHIKV infection at 5 MOI for 2 h. Post-CHIKV infection, the cells were washed and supplemented with complete RPMI media till the harvesting time point (8 hpi).

### 2.6 Flow cytometry

The expression of intracellular and surface markers was investigated using a flow cytometry-based study as described before (19, 22). Briefly, the cells were scrapped out with a cell scraper at 8 hpi time point and washed with 1X PBS before distribution to microcentrifuge tubes. For surface staining, the washed cells were subjected to Fc blocking using Fc blocking reagent (catalog number: 130-092-575; Miltenyi Biotec, Germany) as per the manufacturer’s protocol. Next, the cells were incubated with antibodies against surface markers for 30 minutes at 4°C in dark. Finally, the cells were washed with FACS buffer (1X PBS, 1% BSA, 0.01% NAN_3_) and acquired immediately in the flow cytometer. TLR4 and the macrophage activation markers such as CD86, MHC-II and CD14 were tested by surface staining using fluorochrome-conjugated monoclonal antibodies and acquired in the flow cytometer. To study the intracellular markers, such as CHIKV-E2, p-NF-κB or total TLR4, the cells were initially fixed with 4% paraformaldehyde (HiMedia Laboratories Pvt. Ltd., India) for 10 minutes at room temperature and washed with chilled 1X PBS two times to remove any remnant paraformaldehyde. The fixed cells were permeabilized with permeabilization buffer (1X PBS, 0.5% BSA, 0.1% Saponin and 0.01% NaN_3_) for 15 minutes at RT followed by blocking with blocking buffer (1X PBS, 1% BSA, 0.1% Saponin and 0.01% NaN_3_) for 30 minutes at RT. Next, the cells were further treated with primary (anti-M-CHIKV-E2, anti-R-p-NF-κB antibodies) and their respective fluorochrome-conjugated secondary antibodies sequentially diluted in permeabilization buffer. For TLR4 staining, the cells were incubated with fluorochrome-conjugated antibody (anti-M-TLR4-AF647) diluted in permeabilization buffer. Finally, the cells were washed and re-suspended in FACS buffer and kept at 4°C in dark till acquisition in the flow cytometer. The intracellular cytokine staining starter kit -Mouse (catalog number: 51-2041-AK; BD Biosciences, USA) and BD Golgistop Solution (catalog number-554724, BD Biosciences, USA) were used as per the manufacturer’s protocol for dual staining of intracellular cytokine (TNF) and CHIKV-E2 protein together. All samples were acquired using BD LSRFortessa flow cytometer and analyzed by the FlowJo™ software (BD Biosciences, USA). Around ten thousand cells were acquired per sample per experimental set (minimum three biological replicates were performed).

### 2.7 ELISA

The cell-free culture supernatants from different experimental conditions were subjected to cytokine quantification using the BD OptEIA™ Sandwich ELISA kit (BD biosciences, USA) as per the manufacturer’s instructions. Quantification of cytokines was done with respect to the standard curves prepared using the recombinant cytokines with different concentrations at pg/mL, as reported earlier (19, 22, 45).

### 2.8 qRT-PCR and plaque assay

The viral RNA from cell-free culture supernatants was isolated using the QIAamp Viral RNA mini kit (Qiagen, Germany) as performed earlier (40). Briefly, an equal volume of the viral RNA from all experimental conditions was taken for cDNA synthesis using the Primescript™ 1^st^ strand cDNA synthesis kit (Takara Bio Inc, Japan) obeying the manufacturer’s protocol. The E1 gene was amplified using specific primers (CL11F: 5’-TGCCGTCACAGTTAAGGACG-3’, CL12R: 5’-CCTCGCATGACATGTCCG-3’) and the PowerUp™ SYBR™ Green Master Mix (Thermo Fisher Scientific, USA) in Applied Biosystems™ QuantStudio™ 7 Flex Real-Time PCR System (Applied Biosystems, USA) as per the manufacturer’s instructions. The C_t_ values were plotted against the standard curve to determine the corresponding viral copy number as mentioned earlier (19, 40). To study the intracellular CHIKV copy numbers, the total RNA isolation kit (Catalogue number: MB602-50PR, HiMedia laboratories Pvt. Ltd., India) was used to isolate RNA from the cells. 1 μg of total RNA was converted to cDNA followed by qRT-PCR analysis using the above-mentioned kits and reagents. The intracellular viral copy numbers were normalized against GAPDH, the housekeeping gene (Forward:5’-CAAGGTCATCCATGACAACTTTG-3’, Reverse:5’-GTCCACCACCCTGTTGCTGTAG-3’).

The plaque assay was performed using Vero cells to assess the viral titre as per the protocol mentioned earlier (19). In brief, the CHIKV-infected cell-free culture supernatants were used to infect Vero cells. Post-infection, 5% FBS-supplemented DMEM media mixed with 20% methyl-cellulose (catalog number: M0387; Sigma-Aldrich, USA) was laid over the infected cells for 3-4 days. Next, the cells were fixed using 8% formaldehyde (catalog number: M0387; HiMedia Laboratories Pvt. Ltd, India) and stained with crystal violet to determine the plaque forming units (PFU) manually under the white light of trans-illuminator (Vilber Lourmat, France).

### 2.9 Effect of TAK-242 before, during and after CHIKV infection

To investigate the possible anti-CHIKV effect of TAK-242, in specific stages of viral infection, the following experiment was performed in RAW264.7 cells as per the method described earlier (40, 46). Briefly, the TAK-242 treatment was given at different stages of CHIKV infection namely, before CHIKV infection (only pre-incubation), during CHIKV infection, both before and during CHIKV infection (pre+during incubation), post-infection incubation at 0 hpi (the drug was added at 0 hpi) and 8 hpi (the drug was added at 8 hpi). Besides the drug treatment, the CHIKV infection was given in all of the conditions in a similar way as described above i.e., infection was given with MOI 5 for 2 h. The cell culture supernatants were collected at 9 hpi and qRT-PCR was carried out to determine the CHIKV copy numbers.

### 2.10 Viral attachment assay

To investigate whether TAK-242 has any role in CHIKV adsorption during virus infection, a study was performed to quantitate the unbound CHIKV particles as performed earlier (45). Briefly, the RAW264.7 cells were pre-treated with either DMSO or 1μM TAK-242 for 3 h and further subjected to CHIKV infection with MOI 5 for 2 h. After CHIKV infection, the inoculum volume containing unbound virus particles was collected and subjected to plaque assay and/or qRT-PCR to assess the effect of the drug on viral attachment to the cells.

### 2.11 Time of addition experiment

To study the role of TLR4 in specific stages of the CHIKV life cycle, a time of addition experiment was carried out as described earlier (40, 46). To perform the experiment, no drug treatment was given before or during viral infection. Following the CHIKV infection, TAK-242 was added to the cells at different time points post-infection (0,2,4,8,10,12 and 14 hpi). The cell culture supernatants from all of the time points were collected at 15 hpi for the determination of viral titre using plaque assay.

### 2.12 Western blot

The differential expression of viral E2, E1, TLR4 and MAPK proteins pathways was investigated using Western blot analysis as described before (22). Briefly, the cells were scraped from different experimental groups and washed with cold 1X PBS two times before preparation of whole cell lysate using Radio Immuno Precipitation Assay (RIPA) lysis buffer (150 mM NaCl, pH-8, 1% NP-40, 0.5% Sodium deoxycholate, 0.1% SDS, 50 mM Tris). After lysis, the solutions were centrifuged at 15000 rpm for 30 minutes at 4°C and the supernatants were collected. The protein lysates were quantified using Bradford reagent (catalog number: B6916-500 ML, Sigma-Aldrich, USA). 2X Sample buffer (pH-8, 130mM Tris-Cl, 20% glycerol (v/v), 4.6% SDS (w/v), 2% DTT, and 0.02% Bromophenol blue) was mixed with samples in a ratio of 1:1 and 30 μg of total protein was loaded in each well of 10% SDS-PAGE gel. Next, the proteins on the gel were transferred to a PVDF membrane (catalog number: IPVH00010; Millipore, USA) followed by blocking with 3% BSA (catalog number: MB083; HiMedia Laboratories Pvt Ltd, India). Then, overnight primary antibody incubation was performed using different antibodies like the total and phospho-p38 and SAPK-JNK (1:1000), GAPDH and Beta-Actin (1:2000) and CHIKV-E2 (1:1000). The blots were thoroughly washed five times with 1X tris buffer with 0.1% Tween-20 (TBST) and corresponding anti-Mouse and Rabbit HRP conjugated secondary antibodies (catalog number: 31430 and 31460 respectively; Invitrogen, USA) were probed for 2 h at RT. The blots were washed three times with 1X TBST and the images were captured using the ChemiDoc XRS^+^ imaging system and analyzed by the Image Lab software (Bio-Rad, USA).

### 2.13 *In silico* analysis

The ZDOCK webserver was used to study the protein-protein interaction. The protein-protein docking is based on the Fast Fourier Transform algorithm that utilizes a combination of shape complementarity, electrostatics and statistical potential terms for predicting the interaction complex (47). The MD2-TLR4 activated complex (PDB ID: 2Z64) was used as the receptor. The CHIKV-E2 structure extracted from the mature envelope glycoprotein complex of CHIKV (PDB ID: 3N41) was used as a ligand. The top-ranked output was visualized by the PyMol software.

### 2.14 Co-immunoprecipitation

For TLR4-E2/E1 interaction study, the cells were lysed with 1X RIPA buffer (the composition is the same as described in the WB section) after viral infection. The lysates were subjected to immunoprecipitation by the Dynabeads^®^ Protein G Immunoprecipitation Kit (Thermo Fisher Scientific, USA) as per the protocol mentioned earlier (22). Briefly, both the mock and CHIKV-infected whole cell lysates were incubated with primary antibody (E2 or E1) and Dynabeads^®^ protein G. The Dynabeads^®^-Ab-Ag complexes were washed, eluted and processed further for Western blot analysis.

### 2.15 Anti-TLR4 blocking assay

The anti-TLR4 blocking assay was performed in the RAW264.7 macrophage cells as per the protocol described elsewhere with little modifications (48). Before pre-incubation with DMSO or TAK-242, either anti-TLR4 antibody (Catalogue number: 48-2300, Invitrogen, USA) or anti-rabbit IgG antibody (Catalogue Number: 2729s, Cell signaling technology, USA) was added to the pre-incubation media at 5μg/ml concentration. The cells with different treatments were preincubated for 3 h. Next, the cells were given CHIKV infection at MOI 5 for 2 h. The cells were harvested at 8 hpi and subjected to flow cytometry and Western blot-based analysis. The cell culture supernatants were analyzed for secretory TNF level using ELISA based method. Here anti-rabbit IgG antibody was used as a negative control to conduct the experiment.

### 2.16 Animal studies

All animal experiments were conducted by following the guidelines of the Committee for the Purpose of Control and Supervision of Experiments on Animals (CPCSEA) of India with the approval of the Institutional Animal Ethics Committee, NISER (1634/GO/ReBi/S/12/CPSCEA) and Institutional Animal Ethics Committee, ILS Bhubaneswar (76/Go/ReBi/S/1999/CPCSEA).

Six to eight-weeks aged male BALB/c and C57BL/6 mice were used to perform isolation of peritoneal macrophages as mentioned earlier with little modifications (49). In brief, 4-5 mice per set of the experiment were injected with 1 ml of 3.8% Brewer’s Thioglycolate solution in the peritoneum cavity. After 3 days of injection, the mice were sacrificed and the peritoneal lavages were collected from the peritoneum cavity using chilled 1X PBS with 2% FBS in a sterile manner. Around 6x10^6^ total cells were plated in each 90 mm cell culture dish. After 24 h of seeding, cells were washed with 1X PBS at RT and further experiments were performed with the adherent monocyte-macrophage population.

*In vivo* mice model work on CHIKV infection was performed in a similar way as mentioned earlier (40, 46). In brief, 8-9 days old C57BL/6 mice were housed under specific germ-free conditions for 2-3 days before experimentation. For CHIKV infected mice group (n=5), 10-12 days old mice were injected subcutaneously with 1x10^7^ PFU of CHIKV-IS at the flank region of the right hind limb. For the mock mice group (n=5), serum-free medium was injected at the same position. For TAK-242 treated group (n=5), (dose:1 mg/kg body weight of mice) the drug was given orally from a day before CHIKV infection to 6 days after infection at every 24 h intervals. The mock and CHIKV-treated groups received an equal volume of serum-free media with DMSO for the same duration of the study. The dose of TAK-242 used in the current study was determined based on previously published data where 3 mg/kg dose was shown to be non-toxic and effective for similar mouse model experimentation (24, 37). Depending on their symptoms, the mice were sacrificed on the 5^th^ or 6^th^-day post-infection (dpi) followed by the collection of blood serum, quadriceps muscles and spleens from the mock, CHIKV infected with solvent (DMSO) or TAK-242 treated mice groups. The serum TNF level was quantified by ELISA-based cytokine assay. The quadriceps muscles and spleen samples were snap-frozen followed by lysis with RIPA buffer for Western blot analysis. To quantitate the viral titre, an equal amount of tissues from each group was homogenized in serum-free RPMI media followed by syringe filtration using 0.22 μM filters. The solutions were further centrifuged and the supernatants were collected for plaque assay. For, the survival curve and clinical score analysis, a similar protocol was followed as mentioned above (n=6 for all three groups). The mice were monitored every day for the tabulation of clinical score and final survival curve analysis for up to 8 dpi and scored according to the phenotypic symptom-based disease outcomes [no symptoms-0, fur rise-1, hunchback-2, one hind limb paralysis-3, both hind limb paralysis-4, death-5] (40, 46, 50).

### 2.17 Statistical analysis

The GraphPad Prism 9 software (GraphPad Software Inc., San Diego, USA) was used for statistical analysis. All comparisons among different groups were performed by either the One-way ANOVA with Tuckey posthoc test or the unpaired t-test. All data were represented as mean ± SEM. All analyzed data are representative of at least 3 independent experiments where *p <*0.05 was taken as statistically significant (ns: non-significant, *p <0.05; ** *p ≤*0.01; ***p ≤0.001; ****p ≤0.0001).

## 3 Results

### 3.1 TLR4 inhibition abrogates LPS-induced macrophage activation and pro-inflammatory responses in the host macrophages, *in vitro*

The previously published literature already reports that TAK-242-driven TLR4 inhibition abrogates the upregulation of LPS-mediated pro-inflammatory responses in the RAW264.7 macrophages as well as in the BALB/c-derived peritoneal macrophages (34). Therefore, the effect of TAK-242 in LPS induced RAW264.7 cells has been studied as the experimental control for the current investigation.

To determine the working concentration of TAK-242, Annexin V-7-AAD staining was carried out in the RAW264.7 cells and peritoneal macrophages from BALB/c and C57BL/6 mice. For, the hPBMC-derived monocyte-macrophage cells, a MTT assay was carried out. The cells were incubated with different concentrations of TAK-242 for 24 h and more than 95% of the cells were found viable at 2µM concentratio n (**Figures S1A–D**). According to the previously studied data, TAK-242 effectively inhibits the upregulation of LPS-driven pro-inflammatory responses at 1µM concentration in the RAW264.7 cells (34). To investigate the effect of TAK-242 against LPS-mediated pro-inflammatory responses, the RAW264.7 cells were pre-incubated with either DMSO or 1μM of TAK-242 for 3 h and further treated with 500 ng/mL LPS for 6 h (44). TAK-242 was found not to affect the cell surface as well as total TLR4 expressions significantly in the mock RAW264.7 cells (data not shown).

As previously reported, the reduction in the cell surface TLR4 and increase in the total TLR4 occurs upon LPS or virus-mediated stimulations (32, 51, 52). The flow cytometry dot plot analysis revealed that the percent positive cells for the total TLR4 were increased during LPS and LPS with TAK-242 treated conditions with respect to mock significantly [66.5 ± 2.22% (Mock) to 89.5 ± 1.59% (LPS) and 85.5 ± 1.68% (TAK-242+LPS)] (**Figure S2A**). However, the percent positive cells for the cell surface TLR4 expression were reduced during LPS or LPS+ TAK-242 treatment [43.4 ± 1.42% (Mock) to 27.6 ± 1.1% (LPS) and 36.1 ± 0.757% (LPS+TAK-242)] (**Figure S2B**), which coincides with previous reports.

Based on LPS mediated TLR4 signaling mechanism (53, 54), CD14, a macrophage activation marker (43), was investigated as one of the TLR4 signaling molecules for the current study. Moreover, inducible activation markers on macrophages such as CD86 and MHC-II were also studied to demonstrate macrophage activation (19). The flow cytometry dot plot analysis of CD14 showed a significant increment during LPS treatment and further reduction upon TAK-242 treatment [16.233 ± 2.44% (Mock) to 25.2 ± 2.97% (LPS) and 21 ± 3.03% (LPS+TAK-242)] (**Figure S2C**). CD86 was found to increase during LPS treatment and reduce further in TAK-242 with LPS treated condition [65.17 ± 1.337% (Mock) to 71.30 ± 1.553% (LPS) and 66.13 ± 1.325% (LPS+TAK-242)] (**Figure S2D**). MHC-II also showed a similar pattern of expression to CD86 under the same experimental conditions [40.07 ± 1.707% (Mock) to 51.07 ± 1.598% (LPS) and 47.33 ± 1.338% (LPS+TAK-242)] (**Figure S2E**).

The p-NF-κB activation-driven upregulation of pro-inflammatory cytokines (such as TNF) is already reported upon TLR4 activation (34, 55). The p-NF-κB expression was increased during LPS treatment and decreased further upon TAK-242 treatment in the LPS-induced cells [14.73 ± 2.153% (Mock) to 37.15.6 ± 3.762% (LPS) and 25.23 ± 2.533% (LPS+TAK-242)] (**Figure S2F**).

Furthermore, the earlier reports have described TLR4-directed upregulation of p38 and JNK-MAPK phosphorylation during LPS-induced pulmonary epithelial hyperpermeability and LPS treatment in human neutrophils respectively in a concentration-dependent manner (56, 57). Western Blot analysis revealed upregulation of TLR4 in both the LPS and LPS with TAK-242 treated conditions with respect to mock [2.328 ± 0.067 fold (LPS) and 2.205 ± 0.25 fold (LPS+TAK-242)] (**Figures S2G, H**). The assessment of phosphorylation of the SAPK-JNK pathway revealed that the LPS induction upregulates p-SAPK-JNK expression during LPS treatment which gets reduced during TAK-242 treatment in LPS induced cells [9.826 ± 0.62 fold (LPS) and 2.573 ± 0.09 fold (LPS+TAK-242)] (**Figures S2G, I**). Similarly, p-p38 expression showed a similar pattern in the LPS and LPS with TAK-242 treated conditions [2.373 ± 0.39 fold (LPS) and 1.044 ± 0.2465 fold (LPS+TAK-242)] (**Figure S2G, J**).

An earlier report has suggested that the upregulation of LPS-mediated pro-inflammatory responses was inhibited in presence of TAK-242 (34). In the current study, ELISA-based quantification of the secretory TNF showed a massive upregulation of TNF due to the LPS treatment and subsequent restoration upon TAK-242 treatment in a significant manner [394.4 ± 17.4 pg/mL (Mock) to 2585 ± 57.69 pg/mL (LPS) and 552.5 ± 13.06 pg/mL (LPS+TAK-242)] (**Figure S2K**).

Altogether, these results infer that TAK-242-directed TLR4 inhibition significantly inhibits the upregulation of the LPS-induced pro-inflammatory responses where TLR4 internalization might have a possible implication.

### 3.2 TLR4 antagonism reduces CHIKV infection in the host macrophages of different origins, *in vitro*

#### 3.2.1 Inhibition of TLR4 abrogates CHIKV infection in the RAW264.7 cells, significantly

Based on our previous reports, where it was established that maximum CHIKV infection occurs at 8 hours post-infection (hpi) time point in the RAW264.7 macrophages, 8 hpi was selected for cell harvesting to carry out all the experiments of viral infection (19, 22). E2, an envelope protein of CHIKV, was taken as a marker to assess CHIKV infection in different host systems (19, 40, 41, 45, 46).

**Figure 1:**
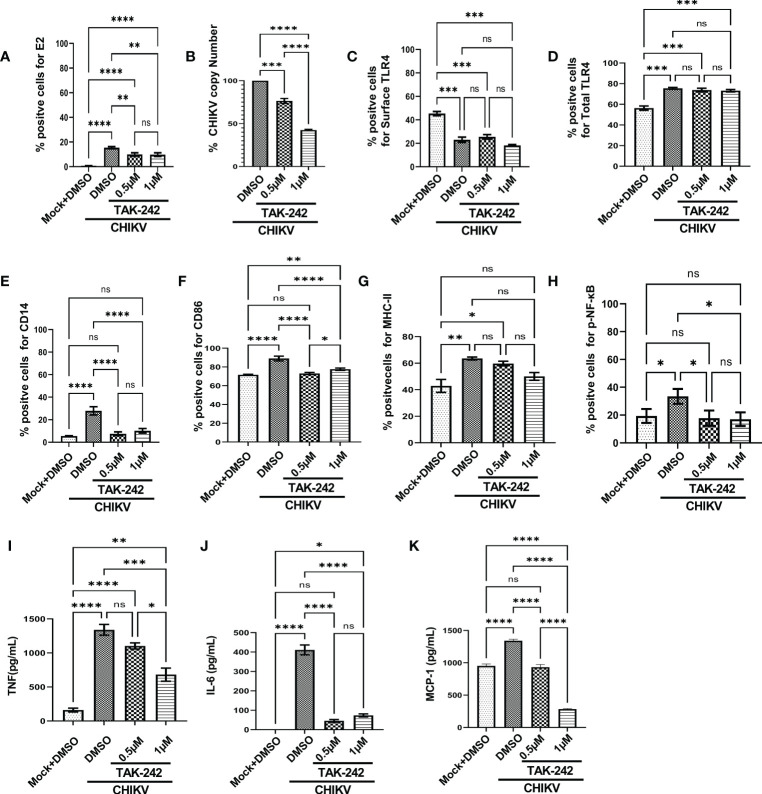
TLR4 inhibition decreases CHIKV infection and pro-inflammatory responses in RAW264.7 macrophage cells, *in vitro*. The RAW264.7 cells were either pre-treated with DMSO or TAK-242 for 3 h before CHIKV infection. CHIKV infection was given at 5 MOI for 2 h followed by the cells were harvested at 8 hpi. **(A)** The bar diagram denotes flow cytometry dot plot analysis based on % positive cells for CHIKV-E2, **(B)** q-RT PCR-based analysis showing decreased CHIKV-E1 copy number in presence of TAK-242. The bar diagrams represent percent positive cells obtained by flow cytometry dot plot analysis for **(C)** surface TLR4, **(D)** total TLR4, **(E)** CD14, **(F)** CD86 and **(G)** MHC-II and **(H)** p-NF-κB expression. **(I–K)** ELISA-based cytokine analysis showing differential expression of TNF-α, IL-6 and MCP-1. Data represent the Mean ± SEM of three independent experiments. *p*< 0.05 was considered as a statistically significant difference between the groups (ns: non-significant, *p <0.05; **p ≤0.01; ***p ≤0.001; ****p ≤0.0001).

To understand the role of TLR4 in CHIKV infection, the TAK-242 treated RAW264.7 cells were infected and harvested at 8 hpi. The cells were subjected to flow cytometry to assess viral infection and macrophage activation. The culture supernatants were used to estimate the viral copy number by qRT-PCR and cytokine levels by ELISA. The reduction of E2 percent positive cells [15.43 ± 0.5175% (CHIKV) to 9.813 ± 0.8411% (TAK-242)] (**Figure 1A**) and significant decrease of corresponding viral copy number [58%] in presence of TAK-242 (1μM) (**Figure 1B**) indicated that TLR4 antagonism reduces CHIKV infection.

In addition, the flow cytometry data showed that the surface expression of TLR4 was reduced upon infection in a significant manner [from 45.33 ± 1.805% to 23.03 ± 2.266%] and it was further decreased nonsignificantly [18.2 ± 0.76%] in presence of TAK-242 treatment (**Figure 1C**). Interestingly, the upregulation of the total TLR4 was observed up on CHIKV infection and in presence of TAK-242 (1μM) [from 56.3 ± 2.066 (mock) to 75.5 ± 3.057 (CHIKV) and 73.2 ± 1.172 (TAK-242)] (**Figure 1D**).

To determine the differential macrophage activation, the percent expressions of CD86, MHC-II and CD14 were investigated in the RAW264.7 cells. It was observed that the percent expression of CD14 was increased in infection and decreased in the presence of TAK-242 (1μM) [from 5.57 ± 0.13% (mock) to 27.9 ± 2.088% (CHIKV) and 10.24 ± 1.157% (TAK-242)] (**Figure 1E**). Similarly, the CD86 expression was found to increase during CHIKV infection which was further reduced in presence of TAK-242 (1μM) [from 71.67 ± 0.29% (mock) to 89.03 ± 1.467% (CHIKV) and 77.6 ± 0.7234% (TAK-242)] (**Figure 1F**). The MHC-II expression was found to be upregulated during CHIKV infection significantly and reduced nonsignificantly during TAK-242 (1μM) treatment [from 42.87 ± 4.889% (mock) to 63.53 ± 1.12% (CHIKV) and 50.07 ± 2.896% (TAK-242)] (**Figure 1G**). Therefore, the data indicate that TLR4 antagonism might reduce CHIKV-mediated macrophage activation.

The level of p-NF-κB was determined by flow cytometry to assess the effect of TAK-242 in TLR4 signaling during CHIKV infection. It was observed that CHIKV infection resulted in an increase of p-NF-κB which was subsequently decreased upon the TAK-242 (1μM) treatment, significantly [from 19.37 ± 2.87% (mock) to 33.43 ± 3.083% (CHIKV) and 17.03 ± 2.854% (TAK-242)] (**Figure 1H**). As per reports, p-NF-κB activation is directly associated with inflammation (58, 59) and pro-inflammatory cytokines such as TNF, IL-6 and MCP-1 which are already reported to be involved with CHIKV-induced immune activation by us and others (19, 33). Accordingly, TNF was found to increase during CHIKV infection and decrease further upon TAK-242 (1μM) treatment, significantly [from 161.2 ± 28.34 (Mock) to 1340 ± 79.26 pg/mL (CHIKV) to 681.5± 97.3 pg/mL (TAK-242)] (**Figure 1I**). Similarly, secretory IL-6 was found to decrease significantly in presence of TAK-242 (1μM) treatment [from 411.1 ± 25.34 pg/mL (CHIKV) to 73.61± 8.047 pg/mL (TAK-242)] (**Figure 1J**). Additionally, reduced MCP-1 expression was also found upon TAK-242 (1μM) treatment [from 951.6 ± 17.19 pg/mL (Mock) to 1342± 12.85 pg/mL (CHIKV) and 286.2± 4.242 pg/mL (TAK-242)] (**Figure 1K**). The representative flow cytometry dot plots of all of the above-mentioned markers were shown in the supplementary section (**Figure S3**).

Further, the current study aimed to elucidate whether TAK-242-directed TLR4 antagonism promotes reduced activation of macrophages or whether overall macrophage activation is solely dependent on the number/percentage of CHIKV-infected cells. To get a detailed insight, flow cytometry-based ICS cytokine staining analysis of TNF-producing cells was performed in CHIKV-E2 positive cells (**Figure S4**). The treatment with TAK-242 (1μM) decreased the frequency of the E2 positive RAW264.7 cells in a significant manner [15.67 ± 1.477% (DMSO+CHIKV) and 9.49 ± 0.9% (TAK-242+CHIKV)]. Respective E2 populations from TAK-242 untreated and treated groups were further analyzed to determine the frequency and expression of TNF in the aforementioned population. The frequency (% positive cells) of TNF-positive cells in both TAK-242 treated and untreated cells was found to be comparable under the E2-selected (gated) population. Interestingly, the mean fluorescence intensity (MFI) of TNF in the E2-gated cells was reduced significantly, which complies with the ELISA data mentioned earlier. The expression of TNF is possibly decreased due to lowered frequency of the E2-positive cells upon TAK-242 treatment. Taken together, the results suggest that TAK-242-directed TLR4 inhibition reduces the CHIKV infection (around 58%) and pro-inflammatory responses, significantly, in the RAW264.7 cells.

#### 3.2.2 Inhibition of TLR4 abrogates CHIKV infection in the primary mouse peritoneal macrophages, significantly

The study was further extended to the CHIKV-infected peritoneal macrophages obtained from the BALB/c mice. It was observed that the percent E2 positive cells [from 26.73 ± 0.98 to 13.27 ± 0.5840] (**Figure S5A**) and the corresponding viral copy number were reduced [60%] significantly in presence of TAK-242 (1μM) (**Figure S5B**). Flow cytometry-based analysis showed that the surface expression of TLR4 was reduced upon infection and TAK-242 (1μM) treatment, significantly [from 75.87 ± 1.247 to 51.07 ± 0.6360% (CHIKV) and 53.5 ± 0.611% (TAK-242)] (**Figure S5C**). However, the total expression of TLR4 was found to increase during infection and TAK-242 (1μM) treatment in comparison to mock, significantly [from 81.5 ± 1.592 (Mock) to 90.73 ± 1.874% (CHIKV) and 89.15 ± 1.084% (TAK-242)] (**Figure S5D**). Moreover, the CD14 expression was found to increase during CHIKV infection and decrease further upon TAK-242 (1μM) treatment, significantly [from 22.53 ± 0.97 (Mock) to 30.73 ± 0.58 (CHIKV) and 26.37 ± 0.44 (TAK-242)] (**Figure S5E**). The CD86 expression was increased during CHIKV infection and further decreased in the presence of TAK-242 (0.5μM), significantly, although a non-significant reduction was observed in presence of 1μM TAK-242 [from 37.47 ± 0.8 (Mock) to 65.23 ± 1.389 (CHIKV), 55.77 ± 0.67 (0.5μM TAK-242) and 60.93 ± 2.009 (1μM TAK-242)] (**Figure S5F**). Moreover, the MHC-II expression was also reduced upon TAK-242 (1μM) treatment, significantly [from 58.2 ± 1.25 (Mock) to 77.67 ± 0.09 (CHIKV) and 69.6 ± 1.513 (TAK-242)] (**Figure S5G**). Next, the p-NF-κB expression was found to increase during CHIKV infection and decrease further upon TAK-242 (1μM) treatment, significantly [from 29.2 ± 3.351 (Mock) to 52.63 ± 3.973 (CHIKV) and 40.87 ± 2.826 (TAK-242)] (**Figure S5H**). To further validate the total TLR4 level, Western blot analysis revealed a significant increase of TLR4 during CHIKV infection and TAK-242 (1μM) treatment [2.123 ± 0.3 fold (CHIKV) and 2.06 ± 0.16 fold (TAK-242)] (**Figures S5I, J**). In order to estimate the inflammatory responses, the levels of TNF, IL-6 and MCP-1 were determined. TNF was found to increase during CHIKV infection and decrease further upon TAK-242 (1μM) treatment, significantly [from 773.2 ± 62.88 pg/mL (CHIKV) to 398.6± 27.58 pg/mL (TAK-242)] (**Figure S5K**). IL-6 was found to increase during CHIKV infection and decrease further upon TAK-242 (1μM) treatment, significantly [from 5.33± 1.294 pg/mL (Mock) to 1078 ± 147.9 pg/mL (CHIKV) and 186.4± 22.98 pg/mL (TAK-242)] (**Figure S5L**). The MCP-1 expression was found to increase during CHIKV infection and decrease further upon TAK-242 (1μM) treatment, significantly [from 145.4 ± 6.667 pg/mL (Mock) to 2117± 152.8 pg/mL (CHIKV) and 377.5± 76.98 pg/mL (TAK-242)] (**Figure S5M**). The data indicate that the TLR4 inhibition reduces the CHIKV infection (around 60%) and associated pro-inflammatory responses, significantly in the peritoneal monocyte-macrophages obtained from BALB/c mice.

Furthermore, a similar study was carried out using the C57BL/6 mice-derived peritoneal macrophages. The percent E2 positive cells [from 18.6 ± 0.95 to 6.558 ± 0.89] (**Figure S6A**) and corresponding viral copy number were significantly reduced [50%] in presence of TAK-242 (**Figure S6B**). Flow cytometry-based analysis showed a similar kind of change in the surface and total expression of TLR4 upon TAK-242 treatment, significantly (**Figures S6C, D**). It was further noticed that although the CD14 and MHC-II expressions were significantly modulated in a similar way, the CD86 expression showed a nonsignificant decrease in presence of TAK-242 treatment (1μM) (**Figures S6E–G**). Accordingly, the p-NF-κB expression was estimated and it was found to increase during CHIKV infection and decrease further upon TAK-242 treatment (1μM), significantly [from 29.6 ± 1.793 (Mock) to 50.7 ± 0.66 (CHIKV) and 35.03 ± 0.5175 (TAK-242)] (**Figure S6H**). The Western blot analysis revealed significant upregulation of TLR4 upon CHIKV infection and also during TAK-242 treatment (1μM) [1.553 ± 0.08 fold (CHIKV) and 1.489 ± 0.14 fold (TAK-242)] (**Figures S6I, J**). As observed before, TNF, IL-6 and MCP-1 followed a similar pattern, significantly (**Figures S6K–M**), indicating that TLR4 inhibition significantly lowers the CHIKV infection (around 50%) and associated pro-inflammatory responses in the peritoneal monocyte-macrophages obtained from C57BL/6 mice as well.

#### 3.2.3 Inhibition of TLR4 abrogates CHIKV infection in the hPBMC-derived macrophages, significantly

To study the effect of TLR4-mediated regulation of CHIKV infection in the higher-order mammalian system, hPBMC derived adherent macrophage population (97% CD14^+^CD11b^+^ cells) (**Figures S7A, B**) was subjected to infection in the presence and absence of TAK-242 (1μM). The hPBMC-derived adherent populations collected from 3 healthy donors showed around a 52% decrease in the E2 level with TAK-242 treatment (**Figures S7C, D**). Similarly, there was a 32.38% reduction in CHIKV infection after TAK-242 treatment as observed by the plaque assay (**Figures S7E**). To assess the pro-inflammatory responses, secretory TNF level was determined using ELISA, where around 44% reduction was observed in TAK-242 treated condition (**Figure S7F**). Collectively, these data indicate that TLR4 inhibition in the hPBMC-derived monocyte/macrophages may lead to reduced CHIKV infection (around 33%) and associated inflammatory responses.

### 3.3 TLR4 inhibition reduces CHIKV infection driven p38 and SAPK-JNK phosphorylation

**Figure 2:**
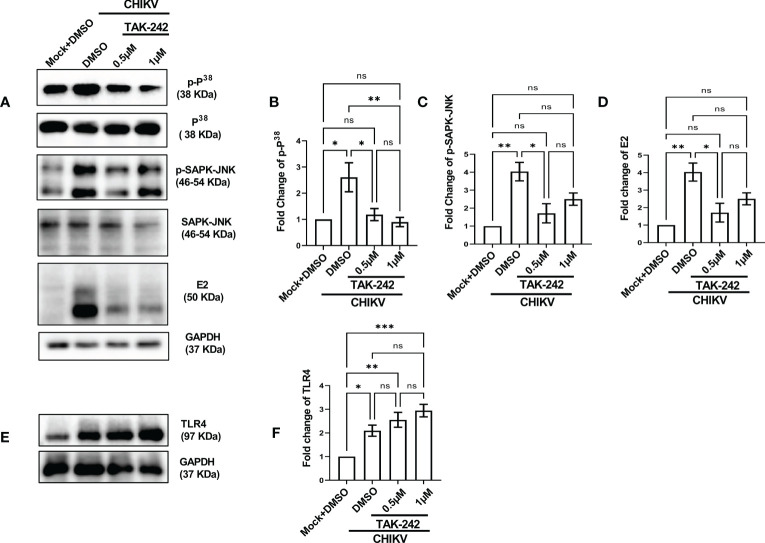
TLR4 inhibition lowers p38 and SAPK-JNK phosphorylation in host macrophages, *in vitro*. RAW264.7 cells were either pre-treated with DMSO or TAK-242 for 3 h before CHIKV infection. The CHIKV infection was given at 5 MOI for 2 h followed by the cells were harvested at 8 hpi. **(A–D)** Western blot analysis showing differential expression of p-P^38^, p-SAPK-JNK, E2 and their quantification normalized against GAPDH, in respective order. (**E, F**) Western blot analysis showing TLR4 expression with the corresponding quantification normalized against GAPDH. Data represent the Mean ± SEM of three independent experiments. *p*< 0.05 was considered as a statistically significant difference between the groups (ns: non-significant, *p <0.05; **p ≤0.01; ***p ≤0.001).

The role of the p38 and JNK-MAPK pathways towards CHIKV infection and inflammation was recently reported (2). To investigate the possible role of TLR4 in MAPK-mediated CHIKV-induced inflammation, differential induction of p-p38 and p-SAPK-JNK-MAPK was observed by Western blot experiment. Significant upregulations of p-p38 (2.9-fold) and p-JNK (4.03-fold) were observed after CHIKV infection in the RAW264.7 cells (**Figures 2A–C**). However, phosphorylation of p38 and JNK was reduced by 4.69 and 1.61-fold respectively following TAK-242 treatment (**Figures 2A–C**). Furthermore, a reduction of the CHIKV-E2 expression (3.27-fold) in presence of TAK-242 (**Figures 2A, D**) was also observed. In correlation with the total expression of TLR4 measured in flow cytometry-based analysis, an increase in the TLR4 expression was found during CHIKV infection (2.09-fold) and TAK-242 treatment (2.9-fold) (**Figures 2E, F**). Collectively, these data indicate that the inhibition of TLR4 might lead to reduced viral infection and induction of the p38, and JNK-MAPK pathways.

### 3.4 CHIKV-E2 and functional TLR4 interaction is necessary for the efficient infection in host macrophages

**Figure 3:**
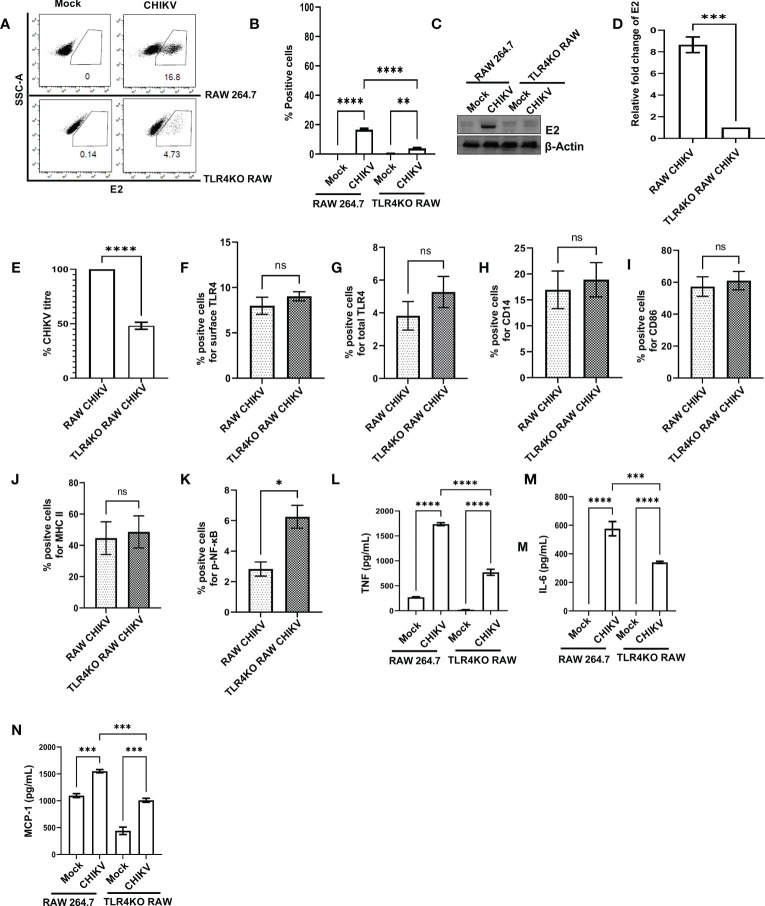
The presence of functional TLR4 facilitates CHIKV infection in host macrophages, *in vitro.* RAW264.7 and TLR4KO RAW cells were subjected to CHIKV infection at 5 MOI and harvested at 8 hpi **(A, B)** The flow cytometry dot plot analysis depicts comparative CHIKV-E2 expression. **(C, D)** Western blot analysis showing comparative E2 level. Normalization of E2 expression was done using β-actin as a housekeeping gene. **(E)** The bar diagram showing comparative CHIKV titre obtained from plaque assay **(F–K)** The flow cytometry dot plot-based bar diagram analysis showing percent positive cells expressing surface TLR4, total TLR4, CD14, CD86, MHC-II and p- NF-κB respectively in mock and CHIKV infected TLR4KO RAW cells. **(L–N)** Bar diagrams depicting ELISA-based TNF-α, IL-6 and MCP-1 quantification respectively in RAW 264.7 and TLR4KO RAW cells. Data represent the Mean ± SEM of three independent experiments. *p*< 0.05 was considered as a statistically significant difference between the groups (ns: non-significant, *p <0.05; **p ≤0.01; ***p ≤0.001; ****p ≤0.0001.

In order to understand the functional association of TLR4 with CHIKV infection, viral infection was performed in the RAW264.7 and TLR4 functional knockout TLR4KO RAW cells. Interestingly, the TLR4KO RAW cell line was used for the current experiment, which is previously reported to show reduced interferon response against SARS-CoV2 specific protein E antigen (36). Therefore, it seems that the functional presence of TLR4 is necessary to implement the SARS-CoV2-specific antiviral responses. The flow cytometry dot plot analysis suggests that the percent E2 positive population in the RAW264.7 cells was reduced in the case of TLR4KO RAW cells (16.60 ± 0.75% to 3.877 ± 0.43%) during CHIKV infection (**Figures 3A, B**). Next, Western blot analysis revealed an around 8.651 ± 0.72-fold decrease of the E2 protein level in the CHIKV-infected TLR4KO RAW cells in comparison to RAW264.7 (**Figures 3C, D**). Assessment of viral titre also showed a 48.11 ± 3.23% reduction in the TLR4KO RAW cells (**Figure 3E**). The total and surface expressions of TLR4 were found to be non-significantly altered (**Figures 3F, G**). Moreover, macrophage activation markers like CD14, CD86 and MHC-II were found to increase in a modest yet non-significant manner during CHIKV infection in the TLR4KO RAW cells in comparison to RAW264.7 (**Figures 3H–J** **)**. However, p-NF-κB was found to increase significantly during CHIKV infection in TLR4KO RAW in comparison to RAW264.7 (**Figure 3K**). To investigate the differential pro-inflammatory responses during CHIKV infection, comparative levels of TNF, IL-6 and MCP-1 levels were quantified by ELISA. These findings report the elevated expressions of TNF, IL-6 and MCP-1 in RAW264.7 by 2.305 ± 0.2219, 1.702 ± 0.1797 and 1.541 ± 0.05658-fold respectively in comparison with TLR4KO RAW (**Figures 3L–N**). Hence, the results obtained from functionally knockout TLR4KO RAW delineate that TLR4 is functionally essential for eliciting the CHIKV-induced pro-inflammatory responses.

**Figure 4:**
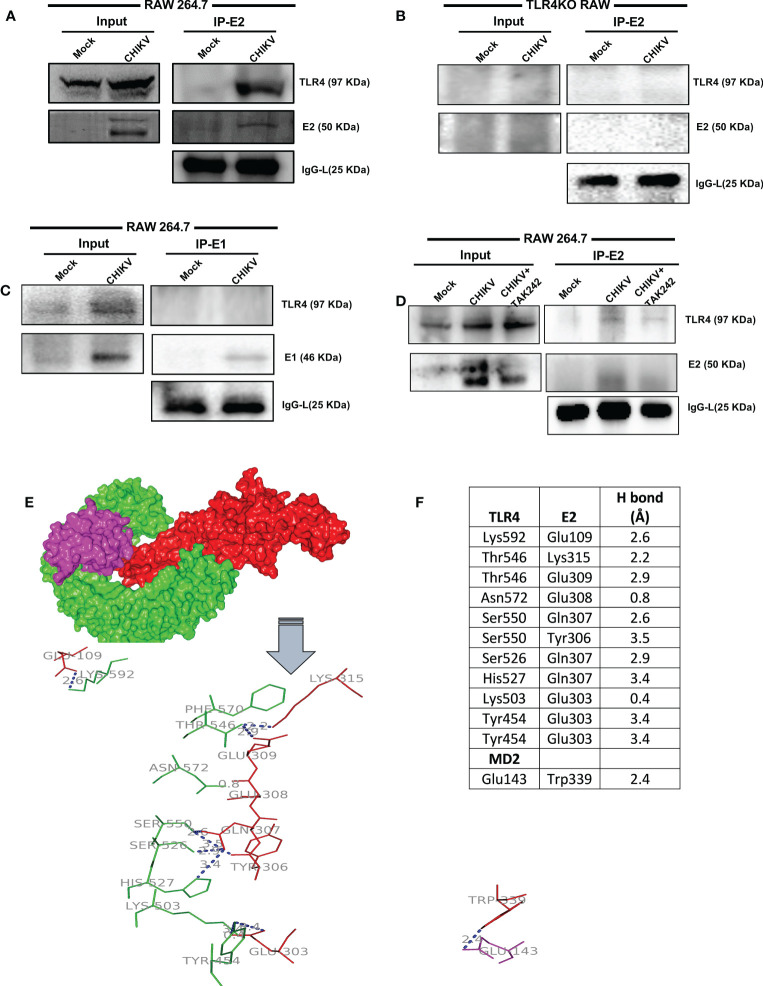
TLR4-E2 interaction facilitates CHIKV infection in host macrophages. The RAW264.7 and TLR4KO RAW cells were subjected to study functional TLR4 and E2 interaction. Both mock and CHIKV-infected cells were processed for immunoprecipitation followed by Western blot analysis. **(A)** For RAW264.7 cells, Western blot analysis showing the expressions of E2 and TLR4 in the whole cell lysate (left), co-immunoprecipitation analysis depicting the interaction of CHIKV E2 and TLR4 (right). **(B)** For TLR4KO RAW cells, Western blot analysis showing the levels of E2 and TLR4 in the whole cell lysate (left), co-immunoprecipitation analysis depicting the interaction of CHIKV E2 and TLR4 (right). **(C)** For RAW264.7 cells, Western blot analysis showing the expressions of E1 and TLR4 in the whole cell lysate (left), co-immunoprecipitation analysis depicting the interaction of CHIKV-E1 and TLR4 (right). **(D)** For RAW264.7 cells, Western blot analysis showing the expressions of E2 and TLR4 in the whole cell lysate (left), co-immunoprecipitation analysis depicting the interaction of CHIKV-E2 and TLR4 (right) in the presence/absence of TAK-242. **(E)** Protein–protein docking analysis reveals probable molecular interaction of MD2-TLR4 (PDB ID: 2Z64) with Chikungunya virus (CHIKV) envelope proteins E2 (PDB ID: 3N41). The protein-protein docking was done in the ZDOCK webserver using MD2-TLR4 as receptor and CHIKV-E2 as ligand **(A)** Interaction complex of MD2 (magenta) and TLR4 (green) with E2 (red). The polar interactions are labeled (blue) in the line diagram. **(F)** The residues involved in polar interactions between CHIKV-E2 and TLR4.

The RAW264.7 cells were infected with CHIKV with MOI 5 and harvested at 8 hpi for further analysis. Co-immunoprecipitation followed by Western blot analysis demonstrated that TLR4 could be pulled with the CHIKV-E2 protein in host macrophages indicating that CHIKV-E2 interacts with host TLR4 (**Figure 4A**). To further validate the results, a study on the interaction of E2 and TLR4 was carried out in the TLR4KO RAW cells under similar experimental conditions. However, no detectable interaction between E2 and TLR4 was observed (**Figure 4B**). To investigate the specificity of the results, the interaction of E1 and TLR4 was studied in the RAW264.7 cells under similar experimental conditions. However, no detectable interaction between E1 and TLR4 was observed (**Figure 4C**). Moreover, less interaction between CHIKV-E2 and host TLR4 was observed in the presence of TAK-242 (**Figure 4D**). The interaction of the extracellular domain of TLR4 and CHIKV-E2 was also validated further by *in-silico* analysis using the mouse TLR4-MD2 complex (PDB ID: 2Z64) and CHIKV structural protein E2 (PDB ID: 3N41) (**Figure 4E**). The analysis showed 12 probable interactions between the amino acid residues of these two structures through molecular docking (**Figure 4F**) suggesting the possibility of TLR4 activation through the interaction of CHIKV-E2 at the extracellular domain of TLR4 that might be required for the efficient viral infection in host macrophages.

**Figure 5:**
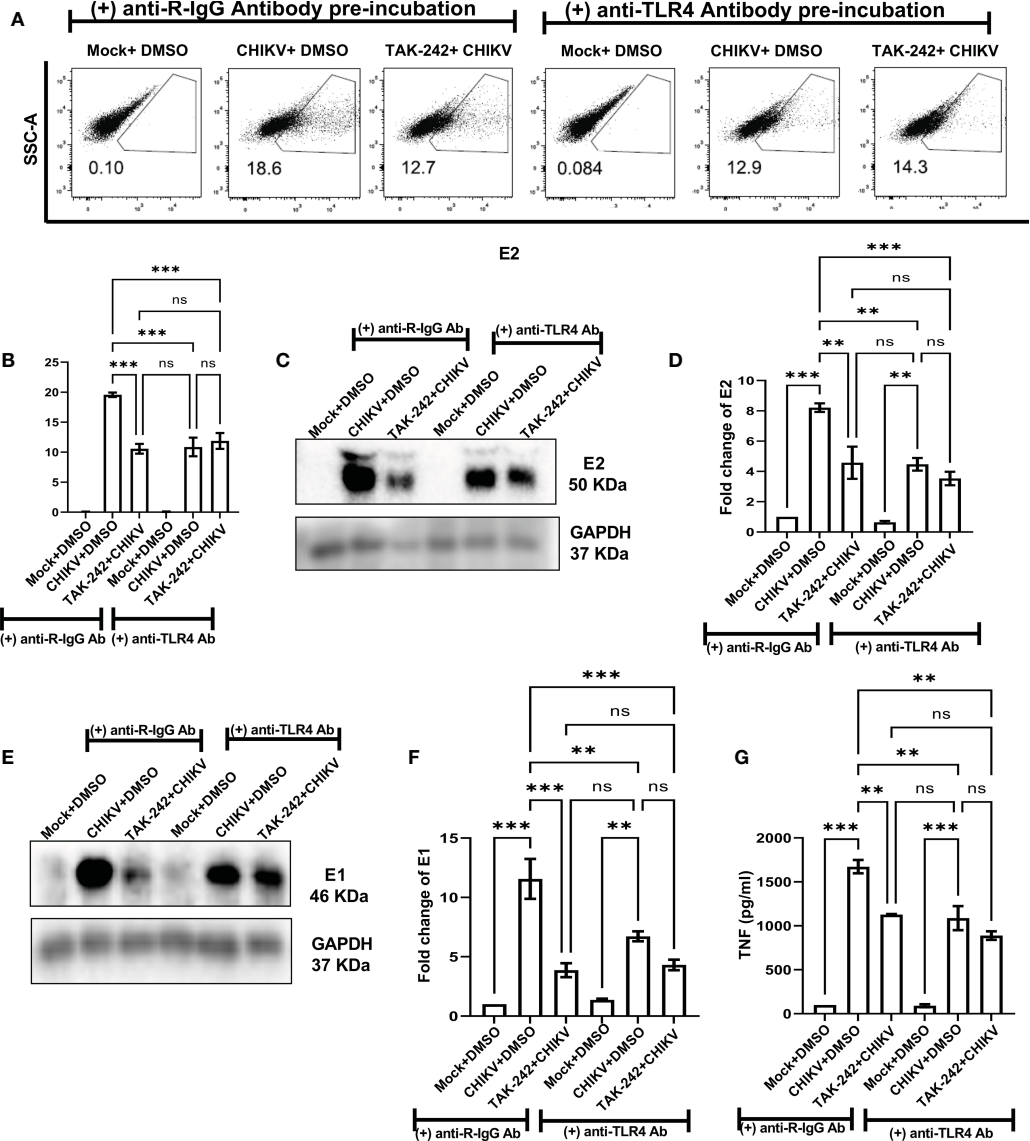
Pre-incubation with anti-TLR4 antibody alleviates CHIKV infection in host RAW264.7 macrophages, *in vitro*. Before pre-incubation of the RAW264.7 cells with either DMSO or TAK-242, the anti-TLR4 antibody or anti-R-IgG antibody was added in the pre-incubation volume in respective conditions at 4 μg/ml concentration and the cells from all conditions were preincubated for 3 h. The CHIKV infection was given at 5 MOI for 2 h and the cells were harvested at 8 hpi. **(A, B)** The flow cytometry dot plot analysis shows comparative CHIKV-E2 levels at different conditions. **(C, D)** Western blot analysis shows E2 expression in different experimental conditions. **(E, F)** Western blot analysis shows differential E1 expression. All densitometric quantifications were performed with respect to GAPDH. **(G)** The bar diagram represents ELISA-based cytokine analysis of TNF.

To further validate the positive regulation of TLR4 on CHIKV infection in host macrophages, the anti-TLR4 antibody-mediated blocking experiment was performed. The flow cytometry-based dot plot analysis revealed a significant decrease in CHIKV infection in the RAW264.7 cells in presence of pre-incubation with the anti-TLR4 antibody. However, in presence of both TAK-242 and anti-TLR4 antibody, CHIKV infection didn’t show any marked change in comparison to only the anti-TLR4 antibody, which might be indicative towards saturation of TLR4 inhibition [from 19.58 ± 0.375% (CHIKV) to 10.57± 0.8168% (TAK-242), 10.87 ± 1.546% (CHIKV+Antibody) to 11.88± 1.316% (TAK-242+CHIKV+Antibody)] (**Figures 5A, B**). Moreover, Western blot analysis revealed the decrease in fold change of CHIKV-E2 level in the anti-TLR4 antibody preincubated condition [from 8.212 ± 0.29-fold (CHIKV) to 4.577± 1.062-fold (TAK-242), 4.469 ± 0.42-fold (CHIKV+Antibody) to 3.53 ± 0.45-fold (TAK-242+CHIKV+Antibody)] (**Figures 5C, D**). Furthermore, the CHIKV-E1 level showed a similar trend of expression to CHIKV-E2 [from 11.56 ± 1.6775-fold (CHIKV) to 3.868± 0.59-fold (TAK-242), 6.725 ± 0.42-fold (CHIKV+Antibody) to 4.315 ± 0.44-fold (TAK-242+CHIKV+Antibody)] (**Figures 5E, F**). Next, ELISA-based cytokine analysis of TNF revealed the reduced level of secretory TNF in presence of the anti-TLR4 antibody-driven pre-incubation, significantly [from 98.84 ± 0.49 pg/ml (Mock) to 1673 ± 75.33 pg/ml (CHIKV), 1127 ± 6.685 pg/ml (TAK-242), 90.68 ± 17.12 pg/ml (Mock+Antibody) 1088 ± 136.6 pg/ml (CHIKV+Antibody) to 889.4 ± 48.26 pg/ml (TAK-242+CHIKV+Antibody)] (**Figure 5G**). Therefore, the anti-TLR4 antibody-driven blocking study reconfirms the possible engagement of host TLR4 as a potential receptor of CHIKV.

### 3.5 TLR4 is required to regulate the CHIKV entry in host macrophages

**Figure 6:**
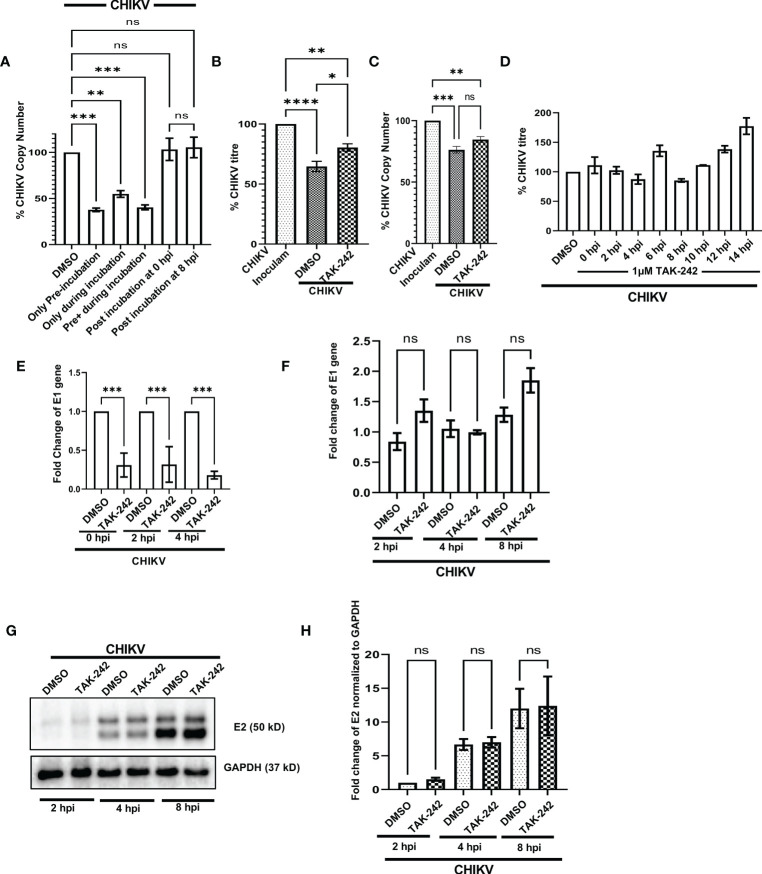
TLR4 promotes viral entry at the early stages of CHIKV infection in host macrophages, *in vitro*. **(A)** TLR4 inhibition before CHIKV infection is most effective to regulate viral copy number at 8 hpi **(B, C)** Viral entry assay in RAW 264.7 cells showing the internalization of around 24% and 11% less virus in TAK-242 treated condition using plaque assay-based viral titre determination and q-RT PCR based viral copy number determination respectively. **(D)** Time of addition assay in RAW 264.7 cells showed no significant decrease in viral infection during post-infection treatment. **(E)** TAK-242 pre-treatment decreases CHIKV copy number in different time points inside the RAW264.7 macrophage cells. **(F)** Post-infection TLR4 inhibition (TAK-242 was added at 0 hpi) does not have a role in CHIKV E1 gene transcription in the RAW264.7 cells. **(G, H)** Post-infection TLR4 inhibition (TAK-242 was added at 0 hpi) does not have a role in CHIKV-E2 translation in the RAW264.7 cells. The densitometry was performed with respect to the corresponding GAPDH expression. Data represent the Mean ± SEM of three independent experiments. *p*< 0.05 was considered as a statistically significant difference between the groups (ns: non-significant, *p <0.05; **p ≤0.01; ***p ≤0.001; ****p ≤0.0001).

To investigate the possible anti-CHIKV role in specific stages of viral infection, the TAK-242 treatment was given in different stages of the CHIKV life cycle as before CHIKV infection (only pre-incubation), during CHIKV infection, both before and during CHIKV infection (pre+during incubation), only during infection (during incubation), post-infection incubation at 0 hpi (the drug was added at 0 hpi) and post-infection incubation at 8 hpi (the drug was added at 8 hpi). It was noticed that the presence of TAK-242 before CHIKV infection (only pre-incubation) and before as well as during CHIKV infection (pre+ during incubation) is most efficient (62% and 59% decrease of CHIKV-E1 copy number, respectively) to regulate the CHIKV infection. Interestingly, a 45% decrease of CHIKV copy number was observed while TAK-242 was added specifically during CHIKV infection only (during incubation), indicating its anti-CHIKV effect. However, no decrease in the CHIKV copy number was observed during the post-infection incubation condition (**Figure 6A**). Therefore, the data suggest that the TAK-242-mediated TLR4 inhibition probably plays a pivotal role in the initial phase of CHIKV infection i.e., the entry and/or attachment stage.

To further confirm whether TLR4 is required in the entry and/or attachment phase of viral infection, TAK-242 (1µM) was added to the RAW264.7 cells before infection for 3 h. Once viral adsorption was over at 37°C, the unbound virus particles (CHIKV in SFM) were collected and subjected to plaque assay and qRT-PCR analysis to determine the viral titre and viral copy number, respectively. It was observed that pre-treatment with TAK-242 resulted in the presence of 24.38 ± 2.302% and 10.86% more CHIKV particles in the wash solution containing unbound virus particles as compared to untreated cells by plaque assay and qRT-PCR-based method respectively (**Figures 6B, C**). Therefore, the data suggest that TLR4 might be required for efficient CHIKV attachment and/or entry in the host macrophages.

In order to confirm whether TAK-242 has any role in a specific phase of the CHIKV life cycle, the “Time of Addition” experiment was carried out as mentioned in the materials and method section. The viral titres were determined for all of the supernatants collected at 15 hpi. The data showed no significant reduction in CHIKV infection at any time point when the drug was added after infection (**Figure 6D**). Hence, the result suggests that TLR4 might not be required for CHIKV once the virus enters inside the host macrophages.

To understand the role of TLR4 in CHIKV replication, E1 mRNA copy numbers were determined inside the cells at different time points after infection. To perform this experiment, the RAW264.7 cells were pre-incubated with TAK-242 (1μM), followed by CHIKV infection at MOI 5 for 2 h with TAK-242 (1μM) and post-infection incubation with TAK-242 (1μM). Next, the cells were harvested at 0, 2 and 4 hpi and subjected to total RNA isolation, cDNA preparation and qRT -PCR analysis of the E1 gene. It was observed that the copy number of the CHIKV-E1 gene was always lower in TAK-242 treated condition inside the cells (**Figure 6E**). This result confirms that TLR4 abrogation leads to the reduced CHIKV replication when TAK-242 is added in pre and pre+ during conditions at different time points as it has been already noticed that post-treatment doesn’t regulate CHIKV infection.

To investigate whether TLR4 inhibition has any role in the transcription of the CHIKV E1 gene, the CHIKV-infected RAW264.7 cells were subjected to post-infection incubation (0 hpi) with TAK-242 (1μM) or DMSO. The cells were harvested at 2, 4 and 8 hpi and subjected to RNA isolation followed by cDNA synthesis and q-RT PCR analysis of the E1 gene to estimate the CHIKV copy number inside the cells. It was found that there is no marked change of the CHIKV-E1 gene in the TAK-242 treated/untreated group at different time points (**Figure 6F**) supporting that post-treatment does not affect the CHIKV transcription.

Similarly, to study the effect of TLR4 inhibition on the translation of E2 protein, the CHIKV-infected RAW264.7 cells were subjected to post-infection incubation (0 hpi) with TAK-242 (1μM) or DMSO. The cells were harvested at 2, 4 and 8 hpi and subjected to Western blot analysis of E2 protein (as representative of CHIKV structural proteins) which depicted no significant difference in the E2 protein level in the TAK-242 treated/untreated group at different time points. (**Figures 6G, H**). These data, therefore, suggest that TLR4 inhibition might not have any role in the viral translation step.

Taken together, all these mechanism-based studies denote that TLR4 might be involved in the CHIKV attachment and entry process in host macrophages and probably doesn’t affect post-entry phases of the CHIKV life cycle.

### 3.6 TLR4 inhibition efficiently reduces the CHIKV infection and inflammation in mice, *in vivo*

**Figure 7:**
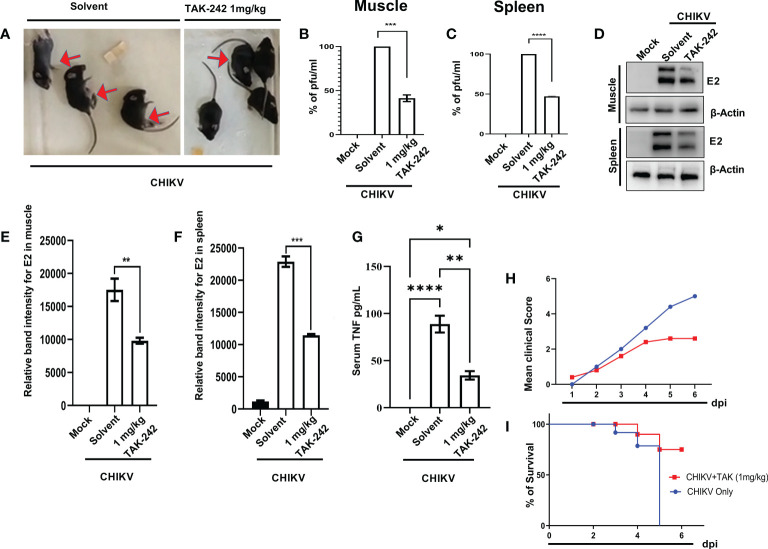
TAK-242 protects mice from CHIKV-driven pro-inflammatory responses and increases survival. 10-12 days old C57BL/6 mice (n=5/group) were injected subcutaneously with 10^6^ CHIKV-IS and treated with TAK242 (dose:1mg/Kg bodyweight of mice) at every 24h intervals up to 4 dpi. After the mice were sacrificed at 5dpi, serum and different tissues were collected for further downstream experiments. To quantitate viral titre, plaque assay was performed using homogenous and filtered tissues sample. For this, an equal amount of quadriceps muscle and spleen were homogenized and filtrated using 0.22µM membrane filter **(A)** The image showing CHIKV-infected mice in the presence and absence of TAK-242 treatment. The arrows indicate mice with impaired limb movement. **(B, C)** The bar diagram shows % of pfu/mL in infected and TAK-242 treated mice muscle and spleen respectively. **(D)** Western blot showing the CHIKV E2 protein in muscle and spleen. Beta-actin was used as a loading control. **(E, F)** The bar diagram showing the relative band intensities of E2 in muscle and spleen respectively in mock, CHIKV and CHIIKV with TAK-242 treated groups **(G)** The bar diagram depicting serum TNF level in mock, infected and TAK-242 treated mice serum **(H)** The line diagram showing the disease symptoms of CHIKV infection which were monitored from 1dpi to 6dpi. **(I)** The survival curve showing the efficacy of TAK-242 against CHIKV-infected C57BL/6 mice (n=6/group). All bar diagrams were obtained through the GraphPad Prism software. Data represent the Mean ± SEM of three independent experiments. *p*< 0.05 was considered as a statistically significant difference between the groups (ns: non-significant, *p <0.05; **p ≤0.01; ***p ≤0.001; ****p ≤0.0001).

The inhibitory role of TAK-242 against CHIKV infection was assessed in 10-12 days old C57BL/6 mice. Interestingly, it was found that TAK-242 treated mice group showed reduced CHIKV-mediated arthritogenic symptoms and impaired limb movements (indicated with an arrow mark in the figure) compared to the only infected group (**Figure 7A**). Following TAK-242 treatment, the viral titre was found to be reduced to 41.26 ± 2.664% and 47.01 ± 0.4225% in the quadriceps muscle and spleen respectively (**Figures 7B, C**). In addition, Western blot analysis revealed the reduction of E2 level to 56.08 ± 2.020% and 50.04 ± 0.6860% in muscle and spleen respectively (**Figures 7D–F**). Moreover, to determine the functional immune response, the serum TNF level was assessed and a reduction of 38.47 ± 2.128% was observed (**Figure 7G**). The clinical score of the TAK-242 treated group of mice showed significantly reduced arthritogenic symptoms as compared to the only infected mice (**Figure 7H**). Additionally, to analyze the survival efficiency of mice in presence of TAK-242, the survival curve was determined and it was found that all of the CHIKV-infected mice died on the 8^th^-day post-infection, while TAK-242 treatment provided 75% better survival during CHIKV infection (**Figure 7I**). Together, the data suggest that TLR4 antagonism effectively reduces CHIKV infection and inflammation and may ensure better survivability (75%) in mice.

## 4 Discussion

TLR4, an important member of the innate immune system, acts as one of the earliest determinants of foreign immunogenic components associated with different sets of pathogens. Starting from its discovery, TLR4 has been known to play a critical role to study the functional aspects of host-pathogen interactions and associated pro-inflammatory immune responses, thus it has evolved as a suitable target for modern-age bio-medical research in the field of rheumatoid arthritis, necrotizing enterocolitis, and inflammatory bowel disease (25, 27, 28). Moreover, the prominent regulatory role of TLR4 has also been explored in the LPS-mediated endotoxin shock and sepsis model in mice using TAK-242 as a probable TLR4 antagonist (24, 37). In the case of LPS-driven TLR4 activation, LPS binding protein (LBP), an extracellular protein, first interacts with LPS present over bacterial outer membrane or in micelle form. A single LPS-LBP complex then interacts with either soluble or the membrane-bound CD14 protein, a co-stimulator of the TLR4 signaling pathway. CD14 acts as a carrier to transfer a single molecule of LPS to MD2 which results in the TLR4-MD2 heterodimer formation which represents the functional LPS receptor. The TLR4-MD2 dimerization occurs to initiate a downstream signaling cascade (60). The LPS induction enhances macrophage activation markers like CD14, MHC-II and CD86 expressions and results in the internalization of cell surface TLR4 (51, 52, 61–64). As reported previously, activation of TLR4 leads to phosphorylation of NF-κB (55) and thus has a direct correlation with inflammation (58, 59). TAK-242, a cyclohexene derivative, has been found to bind selectively to the Cys747 residue of the Toll/interleukin 1 receptor (TIR) domain of TLR4 and inhibits the downstream signaling mechanism (24, 34). According to the previous report, it has been shown that the pre-incubation with 1μM of TAK-242 for 5 minutes can reduce LPS-induced TNF production by 80% in the mouse peritoneal macrophages and the efficacy of the specific anti-inflammatory role of TAK-242 is concentration and time-dependent (34). They have also shown a reduced activation of the NF-κB pathway upon TAK-242-mediated TLR4 inhibition (34). Therefore, the effect of TAK-242-mediated TLR4 inhibition has been simultaneously investigated in the LPS-induced pro-inflammatory model as an experimental control of the current study. Additionally, the re-emergence of CHIKV is considered as one of the global public health threats especially due to the unavailability of possible anti-CHIKV drugs or vaccine to date. The literature on CHIKV infection and pathogenesis report on pro-inflammatory cytokine burst in the host immune system (19). Hence, the current study is intended to explore the involvement of TLR4 during CHIKV infection and associated pro-inflammatory responses.

Earlier studies have already reported that the macrophages could be infected with CHIKV, both *in vivo* as well as *in vitro*, and thus may generate a huge pro-inflammatory cytokine burst (19, 22, 65, 66). The published literature on both mice and macaque models showed that macrophages are one of the immune cells which get recruited at the site of inoculation and generate strong immune responses by pro-inflammatory cytokine release, which might be associated with the CHIKV-induced arthritis, myositis and tenosynovitis (67, 68). CHIKV has already been reported to persist for several months or even years within macrophages and may reappear to cause disease symptoms (65). Therefore, investigating the viral infection-mediated host immune modulation in macrophages might give detailed insight into CHIKV persistence and associated future therapeutic strategies.

TAK-242 (Resatorvid), a well-established TLR4-specific drug has currently been used for clinical trials for several inflammatory diseases, for example, severe sepsis (69) and acute alcoholic hepatitis (ClinicalTrials.gov.Identifier: NCT04620148, https://clinicaltrials.gov/ct2/show/NCT04620148). Therefore, TAK-242 has been used to explore the regulatory role of TLR4, if any, during CHIKV-induced pro-inflammatory responses. The current findings suggest that TAK-242-mediated TLR4 inhibition may abrogate CHIKV infection, cellular activation and pro-inflammatory responses in mouse and human macrophages, *in vitro*. It also demonstrates that TLR4 inhibition-mediated decrease of CHIKV infection is driven by p38 and SAPK-JNK phosphorylation. Interestingly, it is found that CHIKV-E2 interacts with TLR4 during infection which is essential for efficient viral infection in host macrophages. The interaction of the extracellular domain of TLR4 and CHIKV-E2 has been further validated by *in-silico* analysis using the mouse TLR4-MD2 complex as the ligand and CHIKV structural protein, E2 as the receptor. The analysis demonstrates 12 probable interactions where Thr546, Ser550 and Tyr454 residues of TLR4 are found to be critically essential to interact with CHIKV-E2, *in silico*. Therefore, the study depicts TLR4 as one of the possible receptors of the CHIKV-E2 protein to facilitate viral infection. Moreover, anti-TLR4 antibody-dependent blocking assay strengthens the role of TLR4 as a possible receptor for CHIKV-E2 and thus TLR4-mediated CHIKV entry in the RAW264.7 macrophages. Furthermore, it has also been observed that TLR4 plays a key role in CHIKV attachment process and thus TLR4 inhibition might lead to an overall decrease in viral titre. The study also suggests that TLR4 inhibition has no role in post-entry stages of viral infection i.e. viral transcription, replication and translation inside the host macrophages. Additionally, the TLR4 antagonism effectively reduces CHIKV infection and inflammation, *in vivo* by reducing the disease score, significantly with improved survival of CHIKV-infected mice. Therefore, the positive regulation of TLR4 on CHIKV infection in different host systems could be associated with the inflammation and viral pathogenesis.

An earlier report on the respiratory syncytial virus (RSV) describes that the functional TLR4 is an essential component to promote viral infection and the infection-induced inflammasome activation, vascular damage, T cell activation, B cell maturation and NK cell activation in mice model (30). Recent studies on SARS-CoV2 imply that TAK-242 mediated TLR4 inhibition significantly abolishes viral spike protein-induced pro-inflammatory cytokine responses in association with the p-NF-κB protein in the murine and human macrophages (31, 70). VP3, a structural protein of the foot and mouth disease virus (FMDV) is already reported to interact and induce TLR4 to promote viral infection and associated inflammation (32). Furthermore, previous reports on the reduction in the surface expression of TLR4 and increase in the total TLR4 upon LPS or virus-mediated stimulation are found to be similar to this current investigation (32, 51, 52). Hence, the current study suggests a positive regulation of TLR4 on CHIKV entry, infection and associated inflammation in the host.

Although this study proposes probable TLR4-mediated CHIKV entry, TLR4 inhibition doesn’t completely hinder viral entry in the host. Therefore, it seems that the possible involvement of other cellular receptor/s (18) to execute viral entry and pathogenesis might be crucial under the current experimental scenario, which is yet to be explored. Moreover, siRNA-mediated gene silencing could be explored as a suitable tool to investigate the detailed role of TLR4 during viral infection.

The *in-silico* study reveals the association of specific amino acids of TLR4-MD2 complex and CHIKV-E2 proteins in the current investigation. Two amino acid residues, Asn572 and Lys503 of TLR4 (PDB ID: 2Z64) have been found to show high-affinity polar interactions (< 2 Å) with Glu308 and Glu 303 of CHIKV-E2 (PDB ID: 3N41), respectively. Furthermore, Thr546, Ser550 and Tyr454 residues of TLR4 and Gln307 and Glu303 residues of CHIKV-E2 protein have been shown to exhibit multiple polar interactions to emphasize their prominent role in terms of CHIKV-TLR4 association. Further, it will be interesting to investigate the role of these amino acid residues in this interaction through mutational studies in future.

In addition to the mice model, earlier reports are also available on the CHIKV-driven pro-inflammatory cytokine burst and associated symptoms in human patient studies, *in vivo* (20, 21). Accordingly, the effect of TLR4 inhibition could be further explored in experimental *in vitro* or *in vivo* setups with CHIKV-infected patient samples. Therefore, the probable efficacy of TLR4 inhibition against CHIKV infection might be explored in higher-order mammalian systems in future.

In conclusion, the current study reveals the possible regulatory role of TLR4 at the attachment as well as entry stages of viral infection *via* interaction with the CHIKV structural protein E2. Therefore, TLR4 could be considered as a potential receptor of CHIKV and a positive regulator of the virus driven pro-inflammatory host immune responses. Considering this regulatory role of TLR4, this current study might have translational implications for designing future therapeutic strategies against CHIKV infection to modulate the disease pathogenesis.

## Data availability statement

The original contributions presented in the study are included in the article/**Supplementary Material**. Further inquiries can be directed to the corresponding authors.

## Ethics statement

The studies involving human participants were reviewed and approved by Institutional Ethics Committee, NISER, Bhubaneswar (NISER/IEC/2022-04). The patients/participants provided their written informed consent to participate in this study. The animal study was reviewed and approved by Institutional Animal Ethics Committee, NISER (1634/GO/ReBi/S/12/CPSCEA) and Institutional Animal Ethics Committee, ILS Bhubaneswar (76/Go/ReBi/S/1999/CPCSEA) under the affiliation of Committee for the Purpose of Control and Supervision of Experiments on Animals (CPCSEA) of India.

## Author contributions

SuC, SoC, and CM conceived the idea and designed the experiments. CM, SD, SaC, SG, and SSK did the wet lab experiments. BS performed the *in-silico* experimentation and analysis. SuC and SoC provided the reagents. SuC, SoC, and CM analyzed and interpreted the results. SuC, SoC, CM, TM, SK, and KT wrote the manuscript and prepared the figures. All authors contributed to the article and approved the submitted version.
