# Supplementary material for: TLR4 is one of the receptors for Chikungunya virus envelope protein E2 and regulates virus induced pro-inflammatory responses in host macrophages
Source: Front Immunol. 2023 Apr 20;14:1139808. doi: 10.3389/fimmu.2023.1139808 (PMC10157217; doi:10.3389/fimmu.2023.1139808)
Supplement: Supplementary file 1 [file DataSheet_1.pdf]

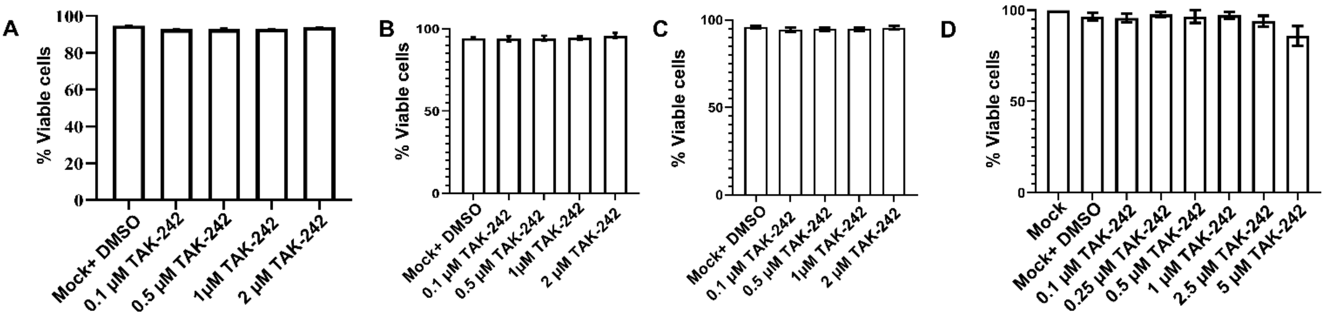

Figure S1: Determination of working concentration of TAK-242 in different host macrophages. (**A**, **B** and **C**) represents Annexin V-7AAD based viability assay in RAW 264.7, C57BL/6 and BALB/c derived peritoneal macrophages, respectively, which is showing > 95% viable cells at 1µM concentration. (**D**) represents MTT assay-based cell viability assay in hPBMC-derived monocyte-macrophage population where >95% cells are viable at 1µM.

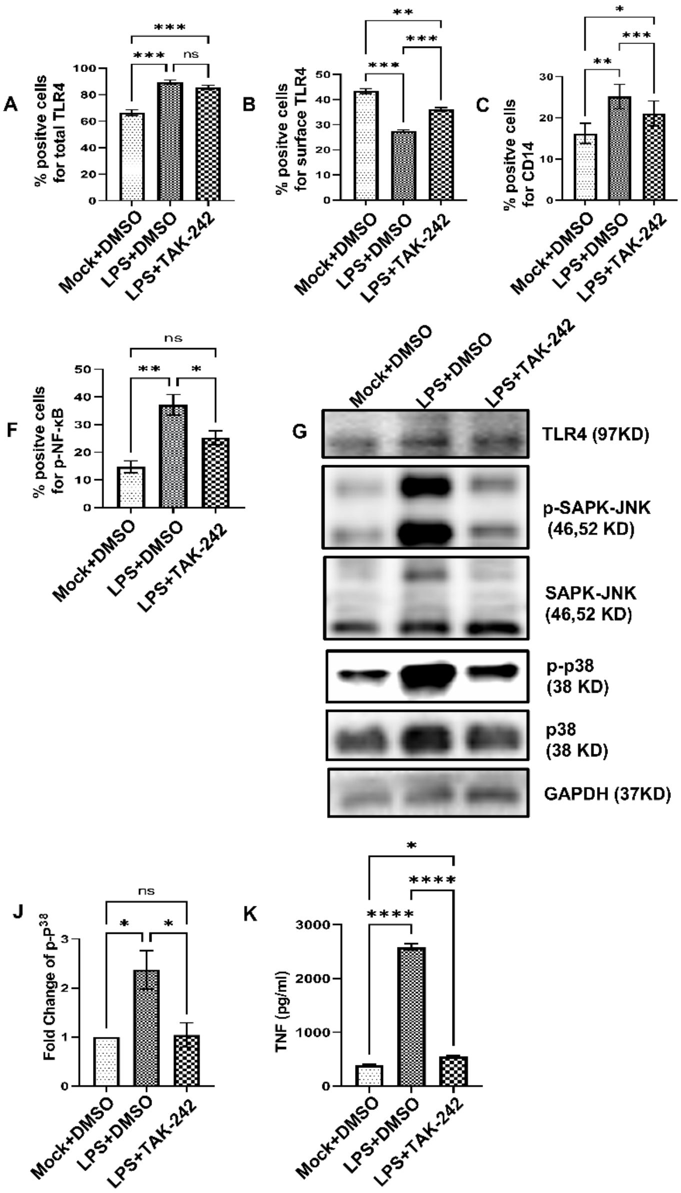

Figure S2: TLR4 inhibition lowers LPS-induced pro-inflammatory responses in RAW264.7 macrophage cells, *in vitro.* The RAW264.7 cells were treated with either DMSO or TAK-242 for 3 h before LPS treatment. LPS treatment was given to the above conditions for 6 h at 1 µg/ml concentration followed by cell harvest. The bar diagrams showing flow cytometry dot plot analysis based on % positive cells for **(A)** total TLR4, **(B)** Surface TLR4, **(C)** CD14, **(D)** CD86 **(E)** MHC-II and **(F)** p-NF-κB. The cells were also subjected to Western blot analysis to show (**G, H**) TLR4, (**G, I**) p-SAPK-JNK and (**G, J**) p-p38 expression**.** All of the proteins were normalized against GAPDH. **(K)** The cell culture supernatants were used for ELISA-based cytokine analysis to show secretory TNF levels. Data represent the Mean ± SEM of three independent experiments. $p< 0.05$ was considered as a statistically significant difference between the groups (ns: non-significant, **p* <0.05; ** *p* ≤0.01; ****p* ≤0.001; *****p* ≤0.0001).

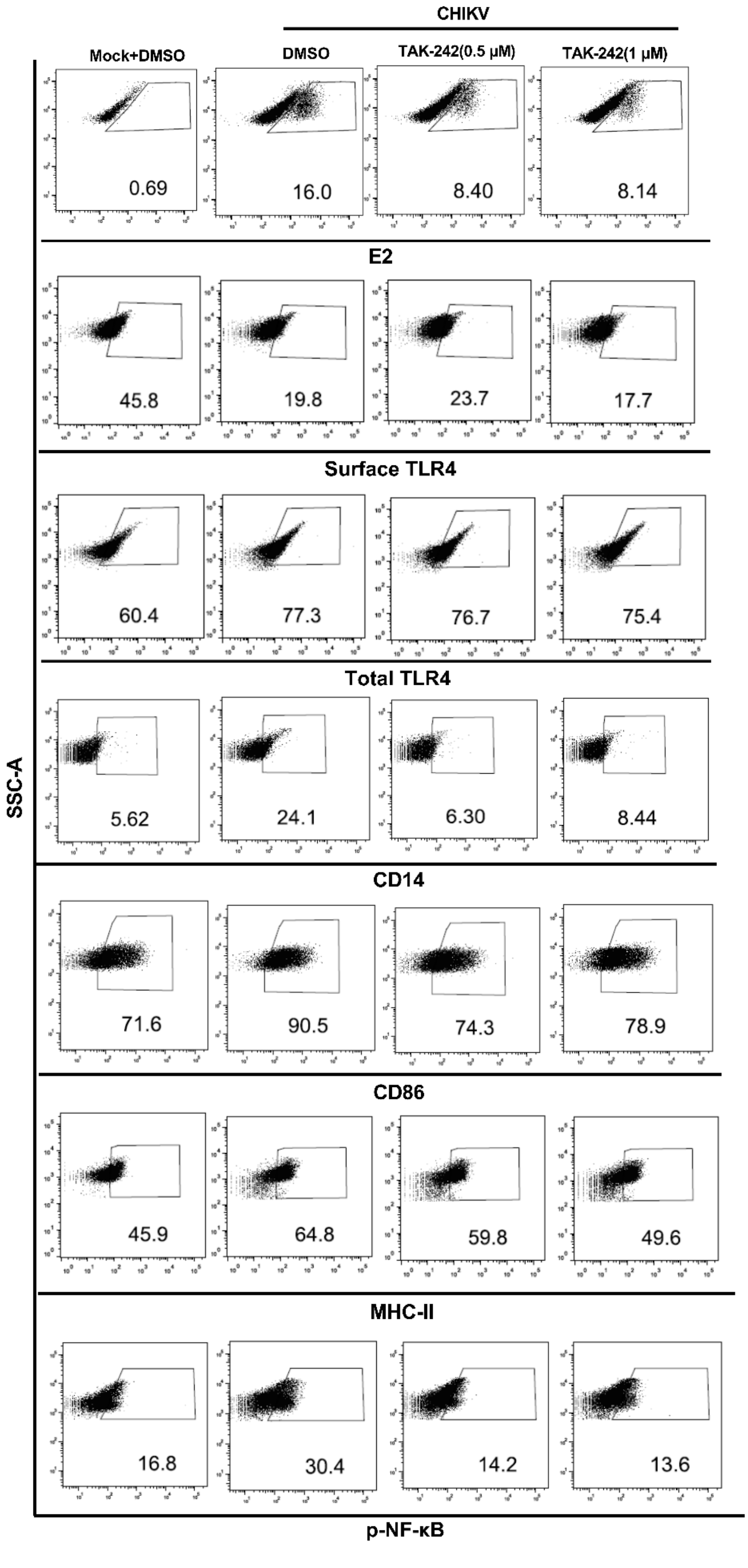

Figure S3: Representative flow cytometry dot plots denoting the percent positive cells for CHIKV-E2, surface TLR4, total TLR4, CD14, CD86, MHC-II and p-NF-κB in the presence or absence of CHIKV and TAK-242 in RAW264.7 macrophage cells.

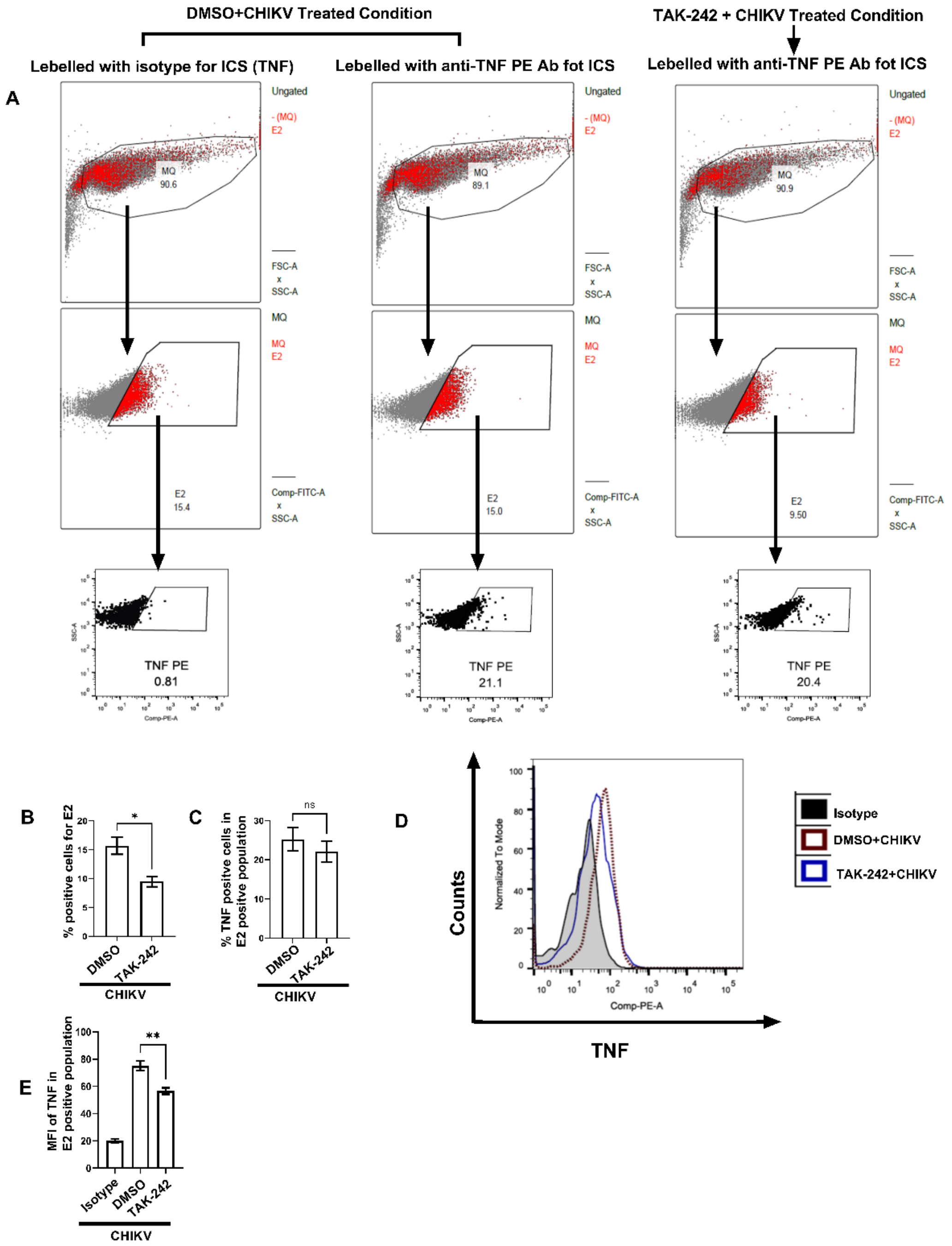

Figure S4: Pharmacological inhibition of TLR4 reduces CHIKV infection and associated proinflammatory response in RAW264.7 cells. ICS staining was performed to characterize the CHIKV-infected population in RAW264.7 cells. The cells were pre-treated with DMSO or TAK-242 3 h before infection. After 4 h post-infection, Golgistop was added to the cells and further incubated for another 4 h. Finally, the cells were harvested at 8 hpi. Dual intracellular staining for E2 and TNF was performed in harvested cells and analyzed via flow cytometry. From the scattered plot, a population devoid of debris was gated and demarcated as **MQ.** E2-positive cells were further gated in the **MQ** population. Furthermore, TNF positive cells were gated in the E2 positive population with respect to their isotype control (**A**) The scattered plots and E2 positive populations of isotype control, DMSO+ CHIKV and TAK-242+CHIKV were represented. Out of the E2-positive population, TNF-PE-positive cells were represented in above mentioned conditions. (**B**) The bar diagram represents % of E2-positive cells (**C**) The bar diagram depicts % of TNF-PE-positive cells within the E2-positive populations. (**D**) The MFI plot denotes the mean fluorescence intensity of TNF-PE positive cells. (**E**) The bar diagram indicates the MFI values of TNF-PE positive cells. The Data represent the mean ± SEM of three independent experiments. $p < 0.05$ was considered as a statistically significant difference between the groups (ns: non-significant, *p <0.05; ** p ≤0.01).

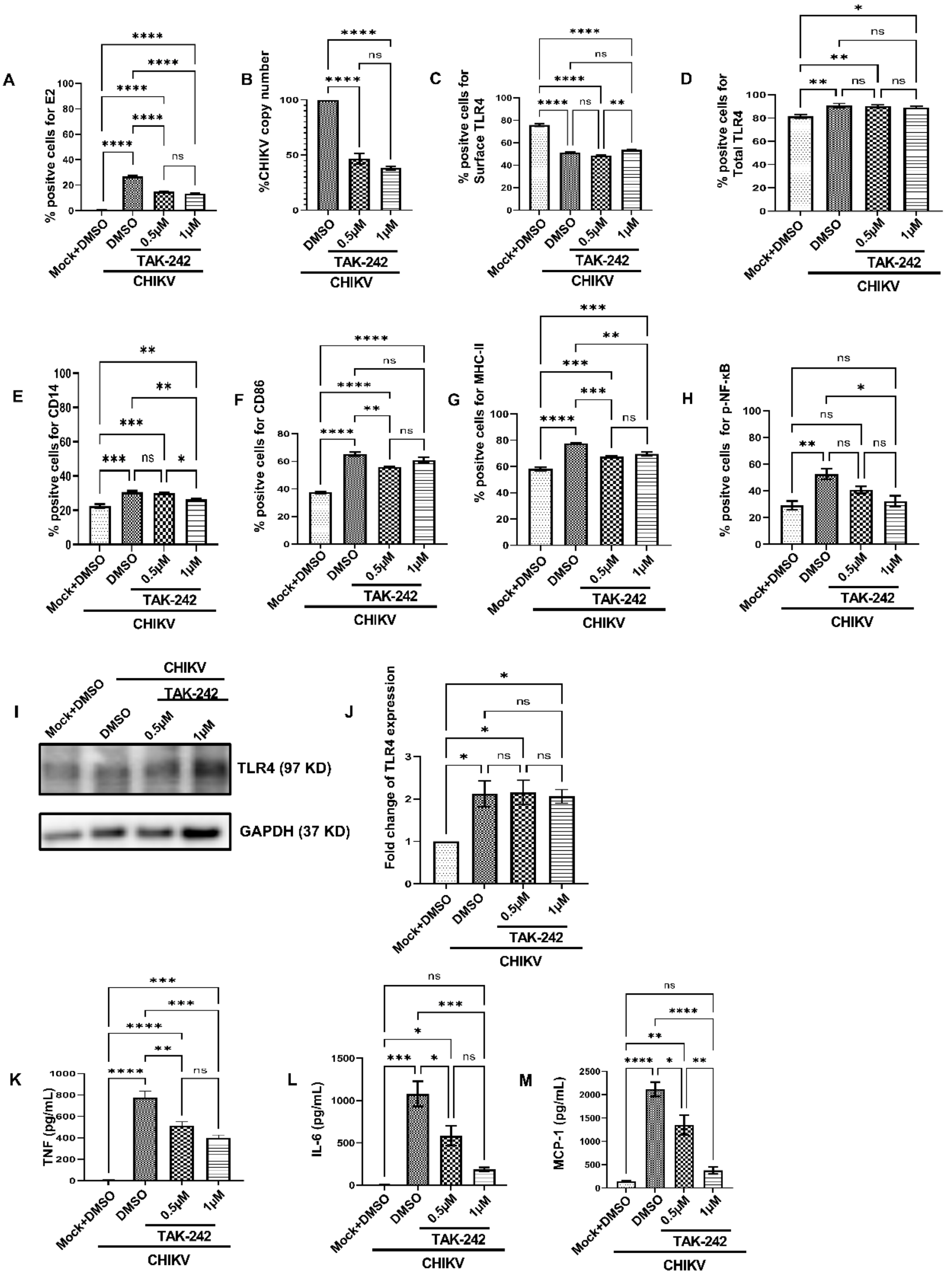

Figure S5: TAK-242 directed TLR4 inhibition reduces CHIKV infection and pro-inflammatory responses in BALB/c derived peritoneal monocyte-macrophage populations, *in vitro*. The cells were either pre-treated with DMSO or TAK-242 for 3 h before CHIKV infection. CHIKV infection was given at 5 MOI for 2 h followed by the cells were harvested at 8 hpi. (**A**) The bar diagram represents flow cytometry dot plot analysis derived percent positive cells for CHIKV-E2. (**B**) q-RT PCR-based analysis representing CHIKV copy numbers. The bar diagrams represent flow cytometry dot plot analysis-based percent positive cells for (**C**) surface TLR4, (**D**) Total TLR4, **(E**) CD14, **(F**) CD86 (**G**) MHC-II and (**H**) p-NF-κB, respectively. (**I, J**) Western blot analysis showing TLR4 level and the densitometric analysis normalized against GAPDH, respectively. (**K-M**) ELISA-based cytokine analysis showing differential expression of TNF-α, IL-6 and MCP-1, respectively. Data represent the Mean ± SEM of three independent experiments. $p < 0.05$ was considered as a statistically significant difference between the groups (ns: non-significant, *p <0.05; ** p ≤0.01; ***p ≤0.001; ****p ≤0.0001).

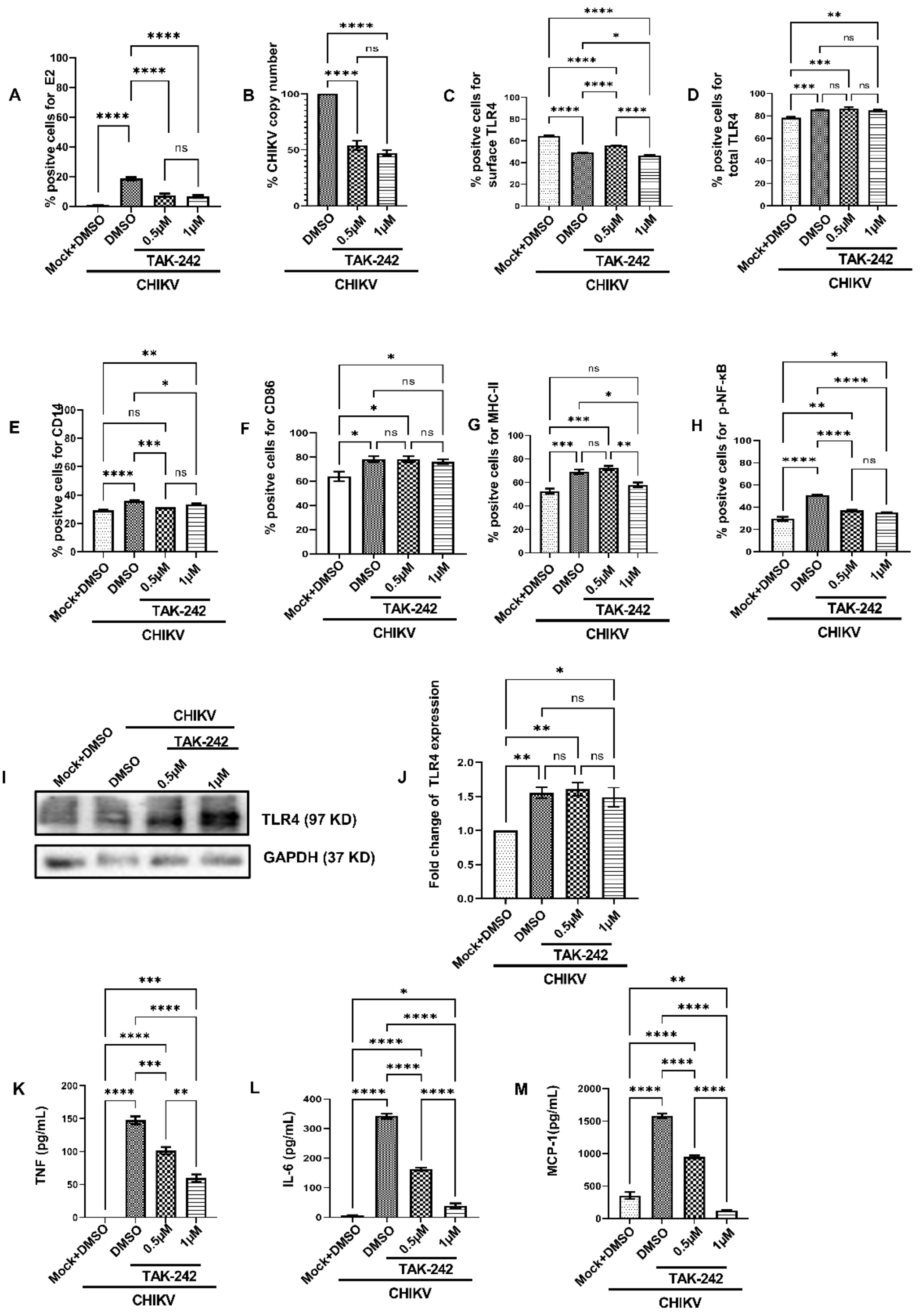

Figure S6: TAK-242 directed TLR4 inhibition reduces CHIKV infection and pro-inflammatory responses in C57BL/6 derived peritoneal monocyte-macrophage populations, *in vitro*. The cells were either pre-treated with DMSO or TAK-242 for 3 h before CHIKV infection. CHIKV infection was given at 5 MOI for 2 h followed by cells were harvested at 8 hpi. (**A**) The bar diagram represents flow cytometry dot plot analysis derived percent positive cells for CHIKV-E2. (**B**) q-RT PCR-based analysis representing CHIKV copy numbers. The bar diagrams represent flow cytometry dot plot analysis-based percent positive cells for (**C**) surface TLR4, (**D**) Total TLR4, **(E**) CD14, **(F**) CD86 (**G**) MHC-II and (**H**) p-NF-κB, respectively. (**I, J**) Western blot analysis showing TLR4 level and the densitometric analysis normalized against GAPDH, respectively. (**K-M**) ELISA-based cytokine analysis showing differential expression of TNF-α, IL-6 and MCP-1, respectively. Data represent the Mean ± SEM of three independent experiments. $p< 0.05$ was considered as a statistically significant difference between the groups (ns: non-significant, *p <0.05; ** p ≤0.01; ***p ≤0.001; ****p ≤0.0001).

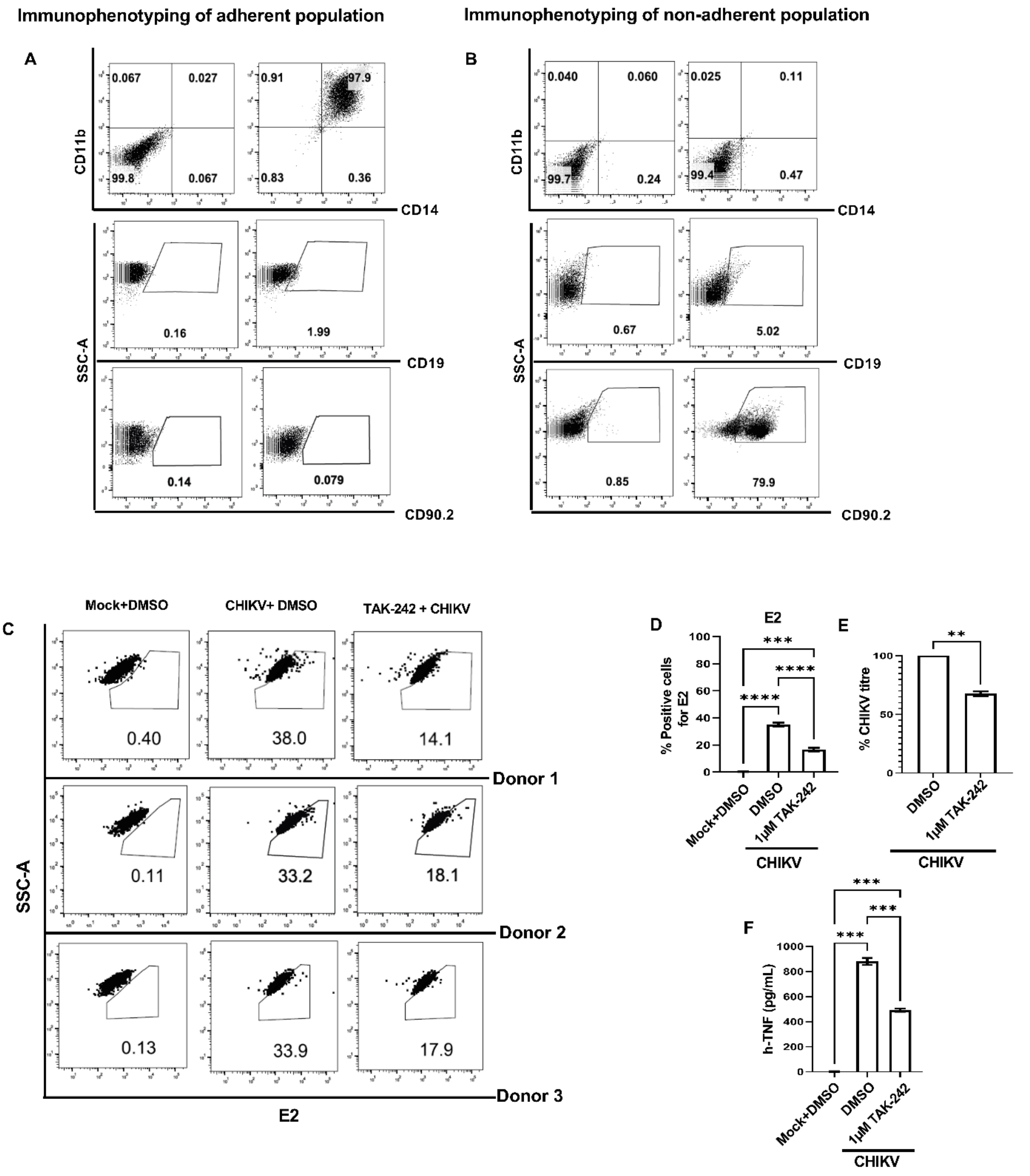

Figure S7: **TAK-242 directed TLR4 inhibition decreases CHIKV infection and pro-inflammatory responses in hPBMC-derived monocyte-macrophage populations, *in vitro*.** The hPBMC-derived adherent monocyte-macrophage cells were either pre-treated with DMSO or TAK-242 for 3 h prior CHIKV infection. The CHIKV infection was given at 5 MOI for 2 h followed by cells were harvested at 8 hpi. (**A, B**) The flow cytometry-based immunophenotyping analysis of adherent and non-adherent populations of the hPBMC-derived myeloid cells. (**C, D**) The flow cytometry dot plot analysis showing percent E2 positive cells under differential conditions. (**E**) Plaque assay-based % CHIKV titre analysis showing viral titre in the presence and absence of TAK-242. (**F**) Cytokine ELISA analysis showing differential h-TNF expression. Data representing Mean ± SEM of three independent experiments with $p < 0.05$ was considered as a statistically significant difference between the groups (ns: non-significant, *p <0.05; ** p ≤0.01; ***p ≤0.001; ****p ≤0.0001).
